# Functional fixedness in chimpanzees

**DOI:** 10.1038/s41598-024-62685-w

**Published:** 2024-05-28

**Authors:** Sonja J. Ebel, Christoph J. Völter, Alejandro Sánchez-Amaro, Katharina A. Helming, Esther Herrmann, Josep Call

**Affiliations:** 1 Department of Comparative Cultural Psychology, Max Planck Institute for Evolutionary Anthropology, Leipzig, Germany; 2 Human Biology and Primate Cognition, Institute of Biology, Leipzig University, Leipzig, Germany; 3 School of Psychology and Neuroscience, University of St Andrews, St Andrews, United Kingdom; 4 Messerli Research Institute, University of Veterinary Medicine Vienna, Medical University of Vienna, University of Vienna, Vienna, Austria; 5 Division of Psychology, Faculty of Natural Sciences, University of Stirling, Stirling, United Kingdom; 6 Department of Psychology, University of Warwick, Coventry, United Kingdom; 7 Department of Psychology, University of Portsmouth, Portsmouth, United Kingdom

**Keywords:** Evolution, Psychology

## Abstract

Differences in the tool use of non-human primates and humans are subject of ongoing debate. In humans, representations of object functions underpin efficient tool use. Yet, representations of object functions can lead to functional fixedness, which describes the fixation on a familiar tool function leading to less efficient problem solving when the problem requires using the tool for a new function. In the current study, we examined whether chimpanzees exhibit functional fixedness. After solving a problem with a tool, chimpanzees were less efficient in solving another problem which required using the same tool with a different function compared to a control group. This fixation effect was still apparent after a period of nine months and when chimpanzees had learned about the function of a tool by observation of a conspecific. These results suggest that functional fixedness in our closest living relatives likely exists and cast doubt on the notion that stable function representations are uniquely human.

## Introduction

The complexity of human tool use is outstanding in the animal kingdom. Humans represent objects not just by their superficial features (e.g., colour) but also by their functions (the terms “objects” and “tools” may be used interchangeably because any object can also be used as a tool)^1^. Even when encountering an object for the first time, humans as early as 24 months old seek information about the object’s function from design features^2,3^. The teleological-intentional stance suggests that humans represent objects as being made for specific purposes by intentional agents^4^. This means that even children assume that objects have been made by humans for a specific function^2^. Early on children start to reason about adults’ actions teleologically, i.e. serving specific purposes^5,6^ and apply this reasoning to elucidate the affordances of tools^3,7^. This indicates that they attempt to deduce the ultimate objective of adults' actions from their initial behaviors and discern the purpose of a tool based on observing others using it or the context in which it is presented.

The focus on function can be so pervasive that it blocks other alternative functions leading to the functional fixedness effect^8,9^. In the so-called “box problem”, participants have to attach three boxes with tacks to a wall, place candles inside and light them with matches^8^. Fewer people solve the task (“attach three candles to the wall and light them”) when the materials are presented inside the boxes compared to lying next to them, which suggests a specific function/use of the boxes. Functional fixedness can be elicited in multiple ways. Priming of the function works by *presenting* task elements in a specific way (as in the previous example) or by *using* the tool for a particular function^8^. Moreover, tools may differ in familiarity, that means, they are either known or new objects, which only get their function by the action performed with them^8^. So far, it is unclear what the effect of different degrees of familiarity is, e.g., if the functional fixedness effect is stronger with more familiar objects. Interestingly, children at the age of six, so relatively late during ontogeny, show functional fixedness when they observe the use of a (new) tool or when they are presented with familiar tools in their functional context (e.g. a spoon in a cup)^10–12^. Functional fixedness is also apparent in cultures with limited tool availability^13^, suggesting it is a universal cognitive bias that inhibits individuals from perceiving alternative uses for familiar objects. At some stage in human evolution, the cognitive capacity to overcome functional fixedness might have emerged concurrent with the development of hierarchical combination of elements for multiple purposes or roles in object manipulation and language as proposed by some researchers^14,15^. This hypothesis suggests that as humans evolved the ability to manipulate objects and develop language, they also acquired the cognitive flexibility to perceive objects beyond their intended function, thereby facilitating problem-solving and innovation.

Non-human great apes use a variety of tools for multiple functions^16,17^. They make and select novel tools to carry out specific tasks^18,19^. However, it is unclear whether apes form enduring representations based on tool function^1,3^. One way to detect if apes, like humans, ascribe endurable functions to objects is to elicit functional fixedness. Studies have shown a lack of tool-use flexibility and behavioral conservatism in chimpanzees^20–25^. For example, in a study with wild chimpanzees, individuals consistently adhered to their familiar method to extract honey from a log with artificial holes^22^. When presented with sticks pre-inserted into the holes, the population that typically used leaf-sponges but not stick tools in their habitual tool-use repertoire removed the sticks but did not re-insert them to access honey. In another study involving chimpanzees living under human care, individuals persisted with the demonstrated dipping technique to access honey from holes at the top of small boxes, even when a more efficient probing technique that opened the boxes was demonstrated subsequently^24^ (for comparable results, see Ref.^25^). Despite these studies showing chimpanzees’ conservative nature and resistance to switch to more efficient techniques, none of these studies has systematically investigated functional fixedness yet. For example, they did not control for potential order effects and novelty of task elements^23^ or could not show that the target behavior was within the repertoire of the studied population^22^. Additionally, no study has tested the effect’s temporal stability.

In particular, our study is one of the first to use a tool with a dual function to investigate whether the usage of the tool for one function interfered with the activation of the other (but see Ref.^26^). A recent study showed increased use of a novel food item as a tool when the food item had not been fed in a pretest, indicating some fixation on the (familiar) use or identity of the object^26^. If functional representations play a prominent role in how apes conceive objects, we would expect that apes become fixated after minimal exposure and with long lasting effects. If they represent superficial features only, they should use tools flexibly regardless of their past functions. Ultimately, elucidating whether apes represent tools by their function is important to shed light onto the nature of object representations and in particular the influence of function as an organizing principle in humans and other animals.

**Figure 1:**
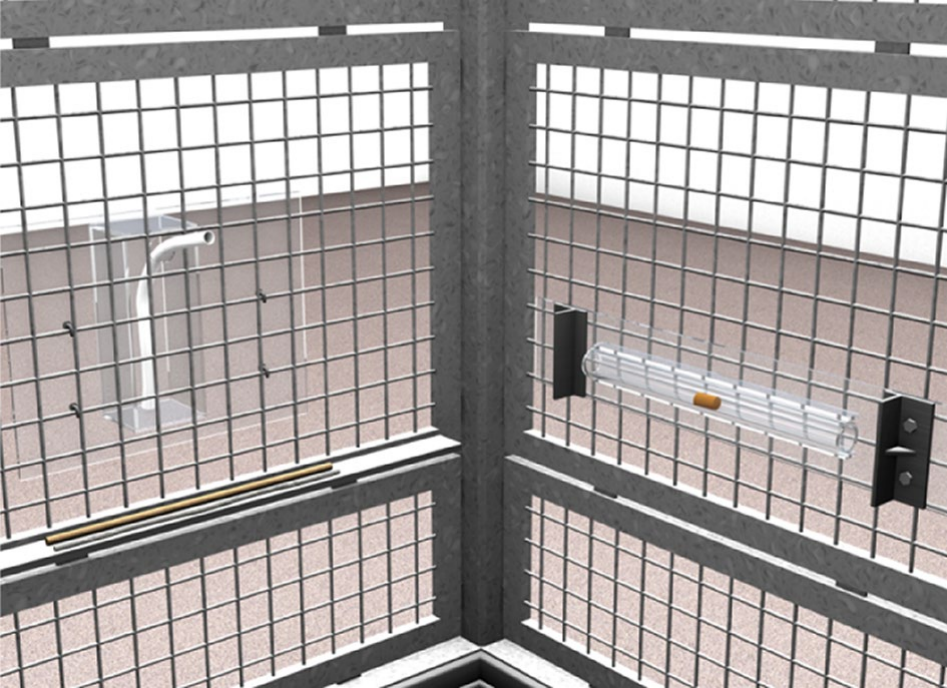
Procedure in Study 1. Participants either drank juice from a drinking container with a hose (experience group) or explored the hose with the empty drinking container present (control group). After this prior experience phase, participants were presented with a horizontal tube with two blockages close to its sides, and had to select the flexible, but rigid hose among three tools (hose, stick, string) to poke out a food reward from the tube. This test assessed if apes become *functionally fixated* on the tool’s previous function by their experience with it.

**Figure 2:**
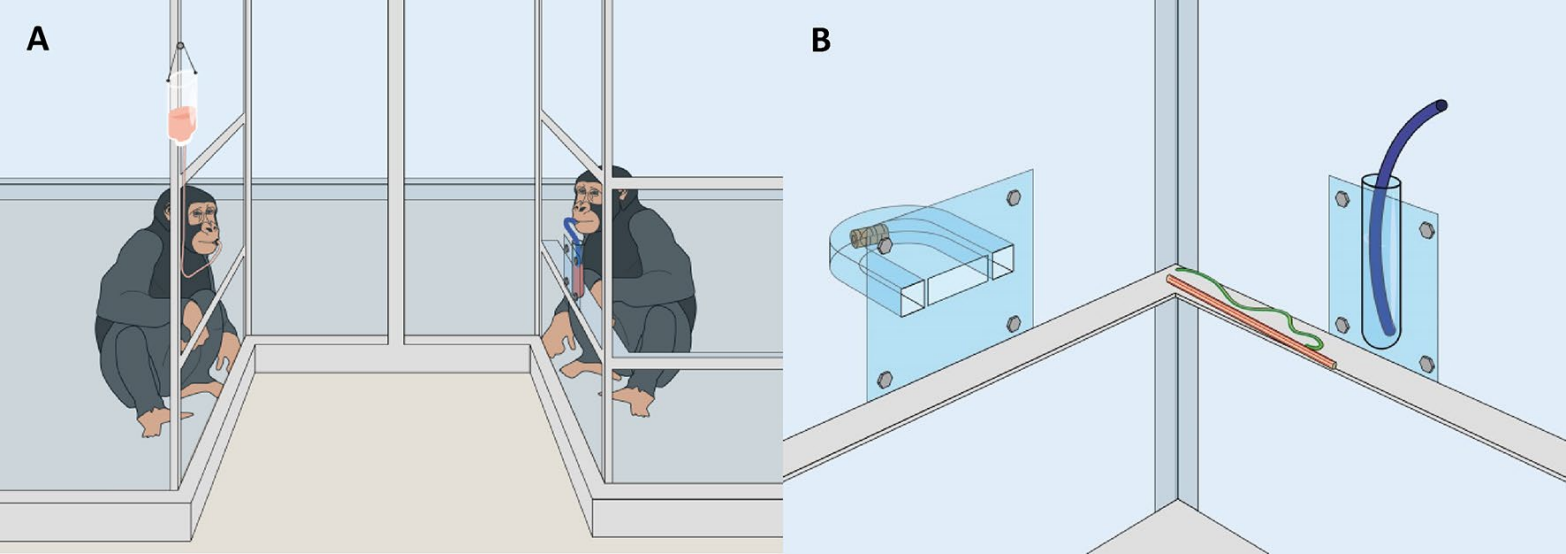
Procedure in Study 2. (**A**) The participant (on the left side) either observed how a conspecific demonstrator (on the right side) drank juice from a drinking container with a straw (experience group) or how she handled the straw without inducing a function (control group). (**B**) After this prior experience phase, the participant was presented with a U-shaped tube. She had to select the flexible straw among three tools (straw, stick, string) to poke out a food reward from the tube.

In Study 1, we presented chimpanzees with a tool-use task to familiarize them with the tool function. Chimpanzees from the experimental group were given a hose to drink juice from a container. Subsequently, we compared these chimpanzees to a naïve control group on a novel tool-use task, which required using the same tool in a new way. To do so, chimpanzees had to select the hose from three tools and use it to push a pellet out of a tube (blockages close to the tube’s exits required the use of a bendable tool, see Fig. 1). Two sessions were conducted close in time and a third one after a delay of nine months to test the stability of the effect. In Study 2, we investigated if chimpanzees exhibited functional fixedness after observing a conspecific using a tool. The conspecific used a (new) straw to drink juice (Fig. 2A). Thereafter, we compared these chimpanzees to a naïve control group on a novel tool-use task that required using the same tool with a new function. Chimpanzees again had to select the straw from three tools to use it to deliver a piece of food from a curved tube (Fig. 2B). We conducted two test sessions close in time.

## Results

### Study 1

**Figure 3:**
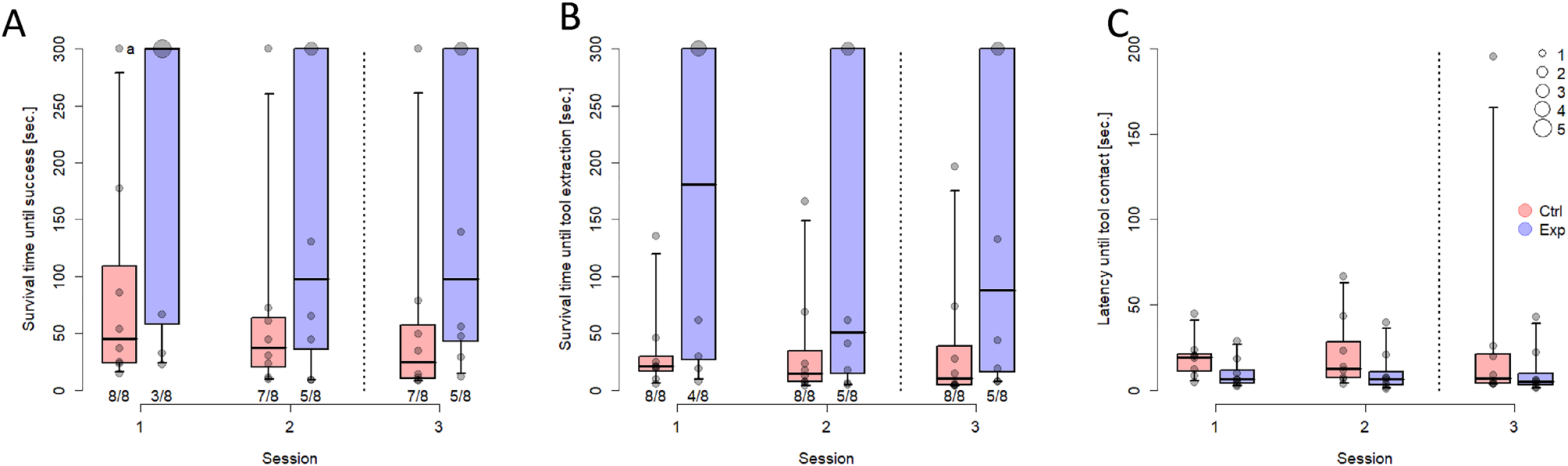
Results of Study 1. (**A**) survival time until success (or until the end of the session at 300 s; survival time is a compound of success and time passed), (**B**) survival time until target tool extraction and (**C**) latency until target tool contact across groups and sessions (median; boxes: 0.25, 0.75; whiskers: 0.025, 0.975). Session 1 and 2 were conducted directly after the prior experience phase, whereas Session 3 was conducted after a delay of nine months. Circles designate individual performance; the circle size is proportional to the represented number of individuals. ^a^One participant succeeded in second 300.

Results indicated that there was no significant difference between the two groups of participants in the overall number of apes solving the task (Fisher’s Exact Test: *p* = 0.200; Exp: 5/8, Ctrl: 8/8). Yet, apes from the experience group took longer to solve the task than the control group (Cox Mixed model; LRT for group: χ^2^ = 7.33, df = 1, *p* = 0.007; see videos in Supplementary Material). There was some indication of apes solving the task faster across sessions; however, this was only a weak trend (LRT for session: χ^2^ = 2.86, df = 1, *p* = 0.091; Fig. 3A). Upon reviewing the plotted results, it became apparent that the pattern remained consistent throughout the second and third sessions, despite the nine-month delay between them. When looking at the time it took them to select the target tool, participants from the experience group took significantly longer to extract the hose from the drinking container than those from the control group (Cox Mixed model; LRT for group: χ^2^ = 8.26, df = 1, *p* = 0.004) and there was a general tendency for apes to extract the target tool faster across sessions (LRT for session: χ^2^ = 3.44, df = 1, *p* = 0.063; Fig. 3B).

We also analysed more closely whether the participants from the experience group noticed the hose (i.e. touched it) and showed any behaviour that indicated that they remembered its previous function (i.e. performed sucking actions). Interestingly, participants from the two groups did not differ in the time it took them to make contact with the hose during the test (LMM; LRT for the full-null-model comparison: χ^2^ = 4.69, df = 3, *p* = 0.196; Fig. 3C). Additionally, there was no difference between the two groups with regard to how frequently they used the hose as the first tool at the test tube (GLMM; LRT for the full-null-model comparison: χ^2^ = 4.40, df = 3, *p* = 0.221; Exp: 5/24, Ctrl: 11/24). However, participants from the experience group sucked more often at the (dry) hose, which was pre-inserted in the empty drinking container during the test, than control subjects (Fisher’s Exact Test: *p* = 0.041; Exp: 6/8, Ctrl: 1/8).

As a concluding point, it's worth noting that we collected additional data for bonobos and orangutans, but due to several dropouts, we were unable to obtain a balanced sample with the additional species (for the results, see Supplementary Material S1). In addition, before conducting Study 1, we conducted two preliminary studies on apes' tool-use fixation using a tool with two functional ends (Prelim A: a brush tool with a pointed end, Prelim B: an L-shaped tool; see Supplementary Material S2). Unfortunately, the results of both studies were inconclusive because too few individuals solved the task overall, resulting in a floor effect.

### Study 2

**Figure 4:**
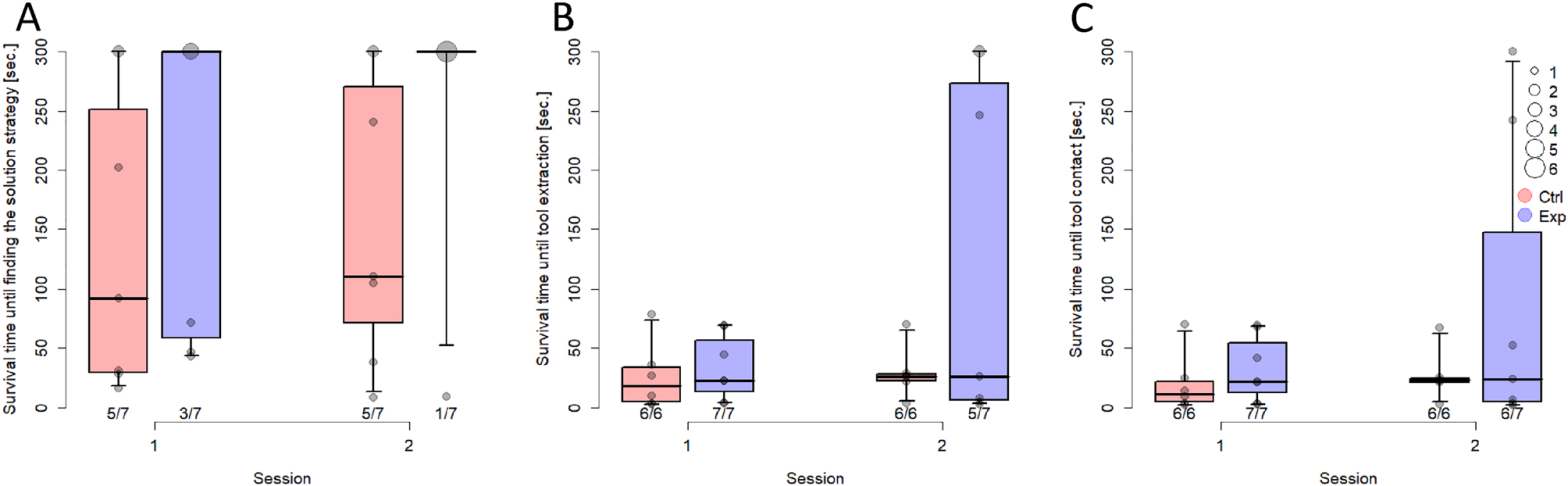
Results of Study 2. (**A**) survival time until finding the solution strategy (or until the end of the session at 300 s), i.e. inserting the hose into the tube without necessarily touching the reward, (**B**) survival time until target tool extraction, and (**C**) survival time until contact with the target tool across groups and sessions (median; boxes: 0.25, 0.75; whiskers: 0.025, 0.975). Circles designate individual performance; the circle size is proportional to the represented number of individuals.

After either observing a conspecific drinking with a straw (experimental group) or not (control group), only two chimpanzees from the control group subsequently used the straw to retrieve the reward from the U-shaped tube. More precisely, while one of them managed to poke the reward out of the tube, the other one only touched the reward with the straw, but failed to push the reward out. Many of the other participants inserted the straw into the tube’s openings, but did not manage to push the straw around the curve of the U-shaped tube, i.e., they did not touch the reward with the tool and this is why this behavior was not coded as success. The tool, due to its own slightly curved shape, had to be oriented in the correct direction to easily and successfully push the reward out of the tube. Due to the overall low success rates, which limited the analysis of group effects, we exploratively coded two additional measurements: finding the solution strategy (inserting the straw into the tube without touching the reward) and time until finding the solution strategy. Participants from the experience group found the solution strategy significantly less often than those from the control group (GLMM; LRT for group: χ^2^ = 5.23, df = 1, *p* = 0.022) and they tended to take longer to find it (Cox Mixed Model; LRT for group: χ^2^ = 3.37, df = 1, *p* = 0.066; Fig. 4A). We also analysed how long it took participants to select the target tool (one participant from the control group had to be excluded from this analysis because her infant took the straw out of the container in both sessions). There was no difference between the groups in the time it took them to extract the straw from the drinking container (Cox Mixed Model; LRT for group: χ^2^ = 0.34, df = 1, *p* = 0.557; Fig. 4B).

We again analysed the time until subjects established the first contact with the target tool (again with one participant less) and did not find a difference between both groups (Cox Mixed Model; LRT for group: χ^2^ = 0.46, df = 1, *p* = 0.505; Fig. 4C). Moreover, both groups did not differ with regard to how often they used the target tool as the first tool at the U-shaped tube in the first session (GLM; LRT for group: χ^2^ = 0.31, df = 1, *p* = 0.576, Exp: 2/7, Ctrl: 3/7) or if they sucked at the straw across sessions (GLM; LRT for group: χ^2^ = 0.31, df = 1, *p* = 0.576; Exp: 3/7, Ctrl: 2/7).

## Discussion

Chimpanzees who had gained experience with one function of a tool were less flexible with this tool thereafter compared to naïve individuals. We found that (a) chimpanzees were vulnerable to functional fixedness, reflected by a longer duration to select the target tool, (b) the fixation could still be identified after a period of nine months, and (c) the effect did not seem to only depend on direct experience with the tool but could also be elicited by observation of the tool’s function. Since we took an exploratory measurement for the analyses in the latter case (due to a floor effect), future studies are needed to confirm functional fixedness through social learning.

As predicted by functional fixedness, chimpanzees who had used a tool with a specific function took longer to use it for a novel function than naive individuals in Study 1. Nevertheless, experience did not prevent most participants from finding the tool’s novel function, reflecting chimpanzees’ general tool-use flexibility. Some authors argue that the best measure of functional fixedness is the latency until tool selection because participants may differ in their abilities to produce the solution^10,13^. Moreover, it is difficult to determine the difficulty of a task in advance if you do not have enough participants for a pilot as it is the case in many comparative studies. This is why it is not surprising that a relatively simple task like pushing a reward out of a tube is solved by most apes, yet, the time it takes them to do so differs across test groups, suggesting a fixation effect on prior use. Interestingly, the experience group was as fast as the control group in touching the target tool (often during sucking attempts), but they took longer to select it. This suggests that they noticed the hose, but did not consider to use it for the novel task, but focused on the distractor tools (a stick and a string). Both groups were equally interested in the task as they readily engaged with the apparatus and the tools (Supplementary Material S1, p.4). Thus, a lack of motivation or excessive distraction with the tool’s drinking function by experienced chimpanzees does not explain the difference between the two groups.

The third test session was conducted after nine months to assess the persistence of the effect over time. Although chimpanzees tended to select the tool and solve the task faster across sessions, the pattern of results remained similar over the three sessions and we did not find a significant interaction between group and session. Thus, functional fixedness remained stable across nine months, which is intriguing given that the apes from the experimental group had only used the hose for drinking five times. This finding is consistent with recent evidence showing a remarkable long-term memory in apes^27,28^. Two open questions for research remain: first, the extent of experience needed to induce functional fixedness, and second, whether the induced strength of fixation interacts with the passage of time.

We found tentative evidence that chimpanzees became functionally fixated after observing a tool’s function in Study 2. This was demonstrated by their taking longer to insert the tool at the correct location of the test tube than naïve individuals. Thus, not only humans, but also apes may learn about object functions by observation and become fixated on these functional representations, a finding that challenges current theories^1,3,4^. Interestingly, Manrique and colleagues have already shown that apes and capuchin monkeys can learn about tool properties by observation^19,29^. This aligns well with wild chimpanzees exhibiting distinct socially learned tool-use cultures that vary across populations^17^. These cultures are maintained through different social learning mechanisms, such as emulation and imitation^30^. Overall, studies suggest that chimpanzees tend to rely on the actions of others to access food and imitate them, particularly in contexts where the causal relations are unclear.^30^ However, in other contexts, they may prefer to pursue their own solution, especially when the end-state is known, a behavior termed emulation.^31^ Thus, it is well conceivable that chimpanzees would exhibit a functional fixedness effect when observing tool use, however, we lack studies directly targeting the role of own experience versus observing a conspecific on the functional fixedness effect.

Despite relating well to the literature, our sample size and the low success rate limit our conclusions from Study 2. One key measure (selecting the target tool) did not reach significance. The participants were familiar with a drinking tool from Study 1, so there might have been a carry-over effect. Moreover, by having participants drink from a drinking device to voluntarily keep them in a good position for observation, performance on the subsequent test could have potentially been affected due to the comparable motor action during observation (namely drinking). Thus, future studies are needed to confirm whether functional fixedness can reliably be found in social scenarios in chimpanzees and, if so, to examine whether such fixation effects differ in strength when they are induced socially or individually.

Functional fixedness has been demonstrated in humans, including populations that use few tools in their everyday lives^13^, and six-year-old children^10,11^. Previous studies showed that apes struggle under specific conditions to use tools with alternative functions^22,23^. Yet, many authors do not consider apes to exhibit functional fixedness or to have enduring function representations of tools^1,3,4^. Our results cast doubt on the idea that apes do not form specific and enduring function representations of tools or categorize objects as tools^32^. In fact, we found a highly similar pattern of results across sessions in Study 1, although one session was conducted after nine months. This result suggests some form of stable object representation.

One might argue that chimpanzees from the experimental group executed their previous motor action with the tool (i.e., sucking) and thus, performed worse than the ones from the control group in Study 1. Accordingly, the fixation effect might be caused by a response conflict. However, such a response conflict could also contribute to functional fixedness in humans: Tool functions are represented by certain action plans that are retrieved automatically when one is presented with the tools. This is potentially also the case in studies in which participants are presented with the tool in its functional context (e.g. spoon inside a cup)^10,11^: The presentation of the tool triggers an action plan that is then rejected because it does not serve the actor’s goal (to investigate chimpanzees’ behavior in a task in which the tool is presented in its functional context, we ran two follow-up studies which were not interpretable due to confounds or too few individuas solving the task,﻿ for further information, see Follow-up B in Supplementary Material S2). Thus, although the precise processes contributing to the effect remain open, experience with the tool clearly had an effect on chimpanzees' tool-use flexibility. The results further question the idea that only humans socially learn about object functions^4,33^. We found at least tentative evidence that chimpanzees exhibit functional fixedness after learning about a tool’s function by observation.

Like all studies, this one faces a couple of limitations: The results are based on a rather small sample size from only one population of chimpanzees, and there is a lack of knowledge about individuals' past experiences. With regard to experience, one might argue that the sample could be biased due to varying levels of experience with tools by the chimpanzees. While we generally consider individuals' experience with tools relevant for tool-use studies, we lack a comprehensive list of their past experiences in the current study. However, we have no reason to believe that our groups were systematically biased by experience, as all of the apes had participated in a variety of tool-use tasks in the past, many across decades, and have access to tool-use enrichment devices in their enclosures. Small sample sizes are a common issue in comparative psychology due to limited access to animals, with many studies dealing with sample sizes of less than ten individuals per group. We addressed this issue as best as we could through pseudo-randomization (matching dyads of chimpanzees by sex, age, and general testing motivation, and randomly allocating them into groups), as well as checking model stability before running the analyses whenever possible. Model stability informs us about the impact of each individual on the results of the model. However, with small sample sizes, there is always the possibility that certain individuals, marked by an unknown common trait, may end up unbalanced in the groups by coincidence. Additionally, we only tested chimpanzees from one population, limiting the generalizability of the results. Therefore, it is important to emphasize the necessity of future replication studies using the same or similar designs, testing larger samples from several groups of chimpanzees to confirm the findings.

Future research is needed to elucidate the precise cognitive underpinnings of functional fixedness in human and apes^1^. Human studies differ a lot in the way in which they introduce tool functions (familiar or novel tools; priming by usage, presentation, observation or verbal instruction). We need to compare experimental setups directly to better understand the impact of these variables. Moreover, future studies could examine how the individual and social dimensions of tool use are related to functional fixedness in humans and apes (see Refs.^34,35^). Finally, it would be interesting to investigate whether there are different levels of functional fixedness in apes depending on how many possible functions an object can have (i.e. the degree of functional fixedness, see^36^)—and whether there is a difference between naturally occurring objects (e.g. a stone found on the ground), objects produced by simple manipulations (e.g. a frayed stick produced by a chimpanzee) and more complex built objects (artifacts, e.g. a spear built by a human) in terms of functional fixedness.

## Methods

### Study 1

#### Participants

The final sample consisted of 16 chimpanzees living under human care (*Pan troglodytes*, 9 females; mean age = 23 years, SD = 12, range = 7–42) housed at Wolfgang Köhler Primate Research Center (Leipzig Zoo, Germany). Additionally, we collected data for four bonobos, one chimpanzee and seven orang-utans. However, due to several drop-outs no balanced sample could be obtained with the additional species (see Supplementary Material S1 for more details).

This study was approved by a joint ethics committee of the Max Planck Institute for Evolutionary Anthropology and the Leipzig Zoo. The experiments were non-invasive, voluntary behavioral tests which strictly adhered to the legal requirements in Germany (German Protection of Animals Act; “Tierschutzgesetz”). The keeping of the animals and research comply with the EAZA Minimum Standards for the Housing and Care of Animals in Zoos and Aquariums and the WAZA Ethical Guidelines for the Conduct of Research on Animals by Zoos and Aquariums. All methods were performed in accordance with the relevant guidelines and regulations. The chimpanzees had water available at all times and were fed normally according to their usual daily routine.

#### Procedure

Beforehand, we had conducted two additional preliminary studies on apes’ tool-use fixation with a tool with two functional ends. However, the results were uninterpretable due to too few individuals solving the tasks (see Supplementary Material S2 for more details). We generally considered the approach with a tool with two functional ends promising, especially given observations from wild chimpanzees distinguishing tool ends^37^. Therefore, we stuck to this general approach but modified it to some extent by using a hose with a dual function as the target tool. With a hose, the possible functions are quite distinct, although there is no longer a difference between the two ends. Instead, two inherently different physical features dictate which actions are possible with it: drinking through the hollow feature and poking with the rigid feature of the tool.

The test consisted of two phases, the prior experience phase and the test phase. In the prior experience phase, the setup comprised a transparent drinking container^38^ with a hole at the front and a piece of hose lying in front of it (same as in Ref.^38^), which apes could use to drink the juice. In the test phase, there was a transparent horizontal tube with two blockages close to its openings (which prevented the insertion of the stick, same as in Ref.^39^) in addition to the empty drinking container. Two additional tools of the same length as the hose were placed in front of the container on a ledge (stick, string), while the hose was inserted into the drinking container (Fig. 1; see Supplementary Material S1).

Participants from the experience group received five drinking sessions on separate days, in which they inserted the hose into the container for drinking juice. Sessions lasted until apes had finished the juice. If they did not use the hose spontaneously, we implemented a three-step scaffolding procedure to facilitate drinking (see Supplementary Material S1). Participants from the control group received two exploration sessions with five minutes each, in which they could explore the hose that was lying in front of the empty (and dry) container. Thus, they experienced the hose but not its function as a drinking tool.

Thereafter, participants from both groups were presented with the test setup, in which they had to extract the pre-inserted hose from the container to poke out a banana pellet from the horizontal tube (Fig. 1). There are two different classical ways to test functional fixedness. One way is to have all participants know the tool’s function and then, one group gets the tool presented in its functional context and one does not^10^. The other way is to have two groups that differ in their experience with the tool and only one knows its function in the test. In this study, we tested a mixture of both versions (only one group associates a function with the tool and the tool was presented in its functional context in the test). We conducted a small follow-up study (Follow-up A) to investigate the role of functional context, but the results were inconclusive (Supplementary file S2). Two of the test sessions were conducted immediately after the prior experience phase on two separate days and the third one after 9 months (mean: 9 months, range 8–10) to assess the temporal stability of the experimental treatment. If apes were successful the first time in their second session, they received an additional session immediately thereafter so that all successful apes had the same amount of experience after the first testing period (results of these additional sessions were excluded from the analyses, but they are reported in the Supplementary Material S1, *N* = 2).

#### Analyses

Success and time until success were coded from the videos to establish if the participants’ problem-solving performance decreased after experience with the tool. Since we were unable to pilot the difficulty of the task, we were aware that a floor effect (i.e., few individuals solving the task) or ceiling effect (i.e., most individuals solving the task) would make it impossible to analyze group effects. Therefore, we deemed it important to also consider additional measurements for assessing functional fixedness. Moreover, some authors consider the latency to select the target tool to be a better measure of functional fixedness anyway because participants may need different amounts of time to solve the problem after tool selection^10,13^. When the data structure allowed it, we conducted Generalized Linear Mixed Models (GLMMs) or Cox Mixed Models (the latter were used for the survival times which in contrast to latencies encompass both the duration of time passed and whether an event has occurred or not; see Supplementary Material S1) to analyse the data. The models comprised group, session, and their interaction and age as fixed effects and the random intercept of subject and the random slope of session within subject as random effects. We established p-values using likelihood ratio tests (LRT). When a model did not converge or was unstable, we used non-parametric tests to establish p-values for the variable group (see Supplementary Material S1).

### Study 2

#### Participants

The final sample consisted of 14 chimpanzees (8 females; mean age = 20 years, SD = 9, range = 8–40), of which 13 apes had already participated in Study 1. These individuals were distributed in a counterbalanced manner regarding their drinking experience in Study 1 when assigned to the two groups in the current study. Two additional female chimpanzees (age: 37 and 44 years) served as demonstrators, as well as two male chimpanzees (age: 13 and 16 years) who had finished the test. We collected data with four additional chimpanzees who were dropped from the study due to a lack of motivation (Supplementary Material S1).

#### Procedure

In the prior experience phase, the setup comprised a drinking device (an infusion bottle with a thin hose leading to a hole in the panel) for the participant that positioned them opposing the demonstrator as well as a drinking container and a blue straw for the demonstrator (Fig. 2A). In the test phase, participants encountered a U-shaped tube containing a banana pellet, the empty drinking device with the blue straw pre-inserted, and two additional tools of the same length (stick, string; Fig. 2B). The target tool, the drinking container and the distractor objects differed in their appearance from those used in Study 1 (Figs. 1, 2; Supplementary Material S1).

During the prior experience phase, the participant and the demonstrator were located in two separate rooms. The participant was positioned in front of a transparent Plexiglas panel where she could drink highly diluted grape juice. Participants in the experience group then observed how the demonstrator entered the room, took the straw from the metal frame, inserted it into the container, drank the undiluted juice and then exchanged the tool with the experimenter. This was repeated twice in three sessions, which were conducted on separate days (6 drinking demonstrations). Participants in the control group observed how the demonstrator freely handled the straw without a food reward present and then exchanged it with the experimenter after 30 s. Apes received half a food pellet for the exchange. Three sessions with two demonstrations each were conducted on separate days (6 handling demonstrations).

During the test phase, participants from both groups received two test sessions that lasted 5 min each. The first test session was conducted on the third day after the demonstrations and the second one on a separate day. Before the first test session, the demonstrator left the test room and the setup was prepared. In the test, participants had to extract the pre-inserted straw from the dry container and poke out a banana pellet from the U-shaped tube, while disregarding the stick and a string (see Supplementary Material S1).

#### Analyses

We coded the same variables and applied the same analyses as in Study 1 (GLMMs and Cox Mixed models where appropriate and GLMs or non-parametric tests if the models were unstable, see Supplementary Material S1) except for the following changes due to the small sample size: The models comprised group and session as fixed effects and the random effect of subject ID as well as the random slope of session within subject ID. We additionally coded and analysed finding the solution strategy (i.e., inserting the straw into the tube without touching the reward) and survival time until finding the solution strategy (see Supplementary Material S1).

### Supplementary Information

Supplementary Information 1.

Supplementary Information 2.

Supplementary Information 3.

Supplementary Video 1.

Supplementary Video 2.

Supplementary Legends.

## Data Availability

The data of the main two experiments is published in the Supplementary Material.
