# Supplementary material for: Functional fixedness in chimpanzees
Source: Sci Rep. 2024 May 28;14:12155. doi: 10.1038/s41598-024-62685-w (PMC11130300; doi:10.1038/s41598-024-62685-w)
Supplement: Supplementary file 1 — Supplementary Information 1. [file 41598_2024_62685_MOESM1_ESM.docx]

**Functional fixedness in chimpanzees**

Ebel, Sonja J.; Völter, Christoph J.; Sánchez-Amaro, Alejandro; Helming, Katharina A.; Herrmann, Esther; Call, Josep

***Supplementary Material***

**Additional information on Study 1 & Study 2**

**Study 1**

**Methods. *Participants.*** In addition to the final sample, we tested twelve apes, four of which dropped out of the study during data collection (Table S1): Two bonobos and one chimpanzee failed to drink during the prior experience phase and one orang-utan observed the solution during the test phase. She could not be separated from her 7-year-old daughter who then solved the task. Since we were dealing with a sample biased with respect to species (bonobos: *N_Exp_* = 0, *N_Ctrl_* = 2; orang-utan: *N_Exp_* = 4, *N_Ctrl_* = 2) and we only had data for a third session in chimpanzees, we conducted the analyses with chimpanzees only. However, we also performed the analyses for the first two sessions with all participants (*N* = 24) and results were comparable to those of the analyses with chimpanzees only.
One chimpanzee and three orang-utans had prior experience using a straw to drink juice^1^. We distributed these individuals equally between the two groups. All apes had previously drunk highly diluted juice from a drinking device as part of eye-tracking studies so that they voluntarily stay in one place^2^. However, the eye-tracking studies did not involve active manipulation of a straw-like tool. The hose used in the current study also differed significantly in appearance from the drinking device mentioned.

***Materials.*** We used a rectangular Plexiglas container (L 10 cm x W 10 cm x H 25 cm; same as in Ref. ^1^) with a hole at the front for insertion of the hose (diameter: 4.5 cm) and a hole at the top for refilling (diameter: 3 cm), which was filled with diluted grape juice (100 ml juice + 300 ml water). It was attached to the mesh from outside the test room. For the test, we used a horizontal Plexiglas tube (L 40 cm, outer diameter: 4 cm) with two blockages 5 cm apart from the openings (same as in Ref. ^3^), a grey plastic hose (L 40 cm, diameter: 1.6 cm) commonly used as cable protection, a wooden stick (L 40 cm, diameter: 1 cm) and a string (L 40 cm x W 0.7 cm x H 0.2 cm).

***Procedure.*** Apes were pseudo-randomly divided into two groups that were balanced as much as possible in terms of species, sex and age. The apes from the experience group received six sessions of a three-step scaffolding procedure (depending on performance, see below). The scaffolding procedure consisted of the following steps. In the first two sessions, the hose was already inserted into the drinking container, but could be freely removed. In the next two sessions, the hose was firmly connected to the drinking container and ended in a narrow plastic tube that the apes knew for drinking from eye-tracking studies. In the next two sessions, they received help from the experimenter who first fed them juice through the hose and then put the hose in the container to let them drink on their own. If apes drank from the hose during the first scaffolding step, they then received a baseline session (i.e., the hose was on the metal frame in front of the container). When the apes drank from the hose during the second or third step, they received a session in which they found the hose loosely attached to the container so that it protruded by about 2 cm (it was held in place by a Plexiglas plate with a hole the size of the hose). If they drank again, they received a baseline session afterwards; if not, they returned to the scaffolding procedure. The criterion for passing the prior experience phase was five successful drinking sessions in the baseline, including sessions of the first scaffolding step with a pre-inserted hose (chimpanzees, *N* = 8: mean = 6.4 sessions, range = 5-12). For one chimpanzee (Fraukje), the hose was inserted into the top hole of the container during the test, which was used to refill the juice because she had drunk juice from this hole during the prior experience phase.

**Table S1.** Participants of Study 1.

| **Participant** | **Species(-Group)** | **Sex** | **Age** | **Rearing** |
| --- | --- | --- | --- | --- |
| Bangolo | Chimp-A | Male | 7 | Mother |
| Kofi | Chimp-A | Male | 11 | Mother |
| Lobo | Chimp-A | Male | 12 | Mother |
| Tai | Chimp-A | Female | 14 | Mother |
| Alex | Chimp-B | Male | 15 | Nursery |
| Lome | Chimp-A | Male | 15 | Mother |
| Bambari | Chimp-B | Female | 16 | Mother |
| Swela | Chimp-A | Female | 20 | Mother |
| Frodo | Chimp-A | Male | 22 | Mother |
| Sandra | Chimp-A | Female | 23 | Mother |
| Hope | Chimp-B | Female | 26 | Mother |
| Daza | Chimp-B | Female | 30 | Unknown |
| Dorien^1^ | Chimp-A | Female | 35 | Nursery |
| Riet | Chimp-A | Female | 38 | Nursery |
| Fraukje | Chimp-A | Female | 40 | Nursery |
| Robert | Chimp-A | Male | 40 | Nursery |
| Frederike | Chimp-B | Female | 42 | Unknown |
| Fimi | Bonobo | Female | 8 | Mother |
| Gemena^1^ | Bonobo | Female | 10 | Mother |
| Luiza | Bonobo | Female | 11 | Mother |
| Kuno^1^ | Bonobo | Male | 19 | Nursery |
| Batak | Orang | Male | 7 | Mother |
| Suaq | Orang | Male | 7 | Mother |
| Raja | Orang | Female | 13 | Mother |
| Padana | Orang | Female | 18 | Mother |
| Dokana^1^ | Orang | Female | 27 | Mother |
| Pini | Orang | Female | 28 | Mother |
| Bimbo | Orang | Male | 36 | Nursery |

^1^ drop outs

***Coding and analyses.*** Success (yes/no), survival time until success (yes/no; elapsed time) and survival time until target tool extraction (yes/no; elapsed time) were our main measurements. Survival time is a composite measure of “event” (yes/no) and elapsed time (either until the event occurred or until the session ended), which allowed unsuccessful participants to be included in the analyses. Additionally, latency until first contact with the tool, first tool use at the tube (hose: yes/no) and sucking attempts on the tool (yes/no) were coded from videos. All analyses were performed in R-3.0.2^4^.
*Reliability.* A first coder (SJE) coded the videos and a second coder (LW) rated 20 percent of the videos from the first two sessions with Solomon Coder^5^. Both coders were in good agreement (Pearson’s correlation; time until success: *r* = 1, *df* = 8, *p* < 0.001; time until tool extraction: *r* = 1, *df* = 8, *p* < 0.001; latency until contact with tool: *r* = 0.989, *df* = 8, *p* < 0.001; Cohen’s Kappa; success: *Κ* = 1, *N* = 10, *p* = 0.002; first tool use: *Κ* = 1, *N* = 10, *p* = 0.002; sucking attempt: *Κ* = 1, *N* = 10, *p* = 0.002).
*Success.* We ran a generalized linear mixed model (GLMM) with a binomial error structure and success as the response (*N* = 48; R package “lme4”)^6^. The model included the interaction of group and session plus age as fixed effects and the random intercept of participant plus the random slope of session within participant as random effects^7,8^. Age was log-transformed, and age and session were standardized to their respective means. We tested the model for stability by removing one level of the random effect at a time. The model showed instability caused by the fact that most participants always scored the same value (i.e., “1” or “0”). We therefore analysed overall success using a Fisher’s exact test (*N* = 16).
*Time until success / Time until tool extraction*. We ran Cox mixed models with the same fixed and random effects structure as the model with success as the response (*N* = 48)^9^. The stability of the models seemed reasonable. We compared the models with corresponding reduced models that included only age and the random effects using a likelihood ratio test (LRT). If the comparisons of the full and the null model revealed significance, we tested the significance of each predictor with LRTs*.*
*Time until contact with tool.* The linear mixed model (LMM) with time until first contact with the tool as response had the same structure and was treated the same as the previous models (*N* = 48); stability seemed acceptable. We assessed the normal distribution and homogeneity of the residuals by plotting the residuals which looked acceptable. We then derived variance inflation factors (function “vif” of the R package “car”) and applied them to a standard linear model without the interaction and random effects; there was no evidence of collinearity ^10,11^.
*First tool use.* We analysed the frequency with which participants used the hose as the first tool on the tube using a GLMM with a binomial error structure (*N* = 48). The model included the same fixed and random effects as in the previous models; stability was acceptable.
*Sucking attempts.* The GLMM with binomial error structure had the same structure as before (*N* = 48), but the model was unstable because most participants repeatedly obtained the same value. We therefore performed a Fisher’s exact test to assess the overall behaviour of the apes (*N* = 16).

**Results. *Time until success.*** The full-null-model comparison revealed significance (Cox Mixed model; LRT; χ^2^ = 10.00, df = 3, *p* = 0.019). The interaction between group and session was not significant and was therefore removed from the model (LRT: χ^2^ = 0.05, df = 1, *p* = 0.832). The results of the model are shown in Table S2.

**Table S2.** Results for the model with time until success as the response.

| **Term** | **Estimate** | **SE** | ***χ^2^*** | ***df*** | ***p*** |
| --- | --- | --- | --- | --- | --- |
| Group(Experience) | -2.313 | 0.993 | 7.33 | 1 | 0.007 |
| Session | 0.417 | 0.251 | 2.86 | 1 | 0.091 |
| Age | -1.056 | 0.508 | ^(1)^ | ^(1)^ | ^(1)^ |

^1^ Age served as a control variable only.

***Time until tool extraction.*** The full-null-model comparison revealed significance (Cox Mixed model; LRT; χ^2^ = 11.71, df = 3, *p* = 0.008). The interaction between group and session was found to be non-significant and was excluded from the model (LRT: χ^2^ = 0.081, df = 1, *p* = 0.775). The results of the model are shown in Table S3.

**Table S3.** Results for the model with time until target tool extraction as the response.

| **Term** | **Estimate** | **SE** | ***χ^2^*** | ***df*** | ***p*** |
| --- | --- | --- | --- | --- | --- |
| Group(Experience) | -2.481 | 0.861 | 8.26 | 1 | 0.004 |
| Session | 0.348 | 0.189 | 3.44 | 1 | 0.063 |
| Age | -1.467 | 0.459 | ^(1)^ | ^(1)^ | ^(1)^ |

^1^ Age served as a control variable only.

***Additional sessions.*** Two participants who solved the task in their second session received an additional session in the first test period (i.e., 4 sessions in total) so that they had the same experience with the task as the other successful participants when they faced it again after nine months (additional sessions – Bangolo: time until success = 15 seconds, time until tool extraction = 10 seconds; Hope: time until success = 8 seconds, time until tool extraction = 4 seconds). Additionally, eight bonobos and orang-utans were tested (results – experience group, *N* = 2: success = 2/4 sessions, mean time until success = 172 seconds, mean time until tool extraction = 62 seconds; control group, *N* = 6, success = 12/13 sessions, mean time until success = 55 seconds, mean time until tool extraction = 43 seconds).

**Exploratory coding: motivation to engage with the task**

**Coding.** To examine whether the two groups would differ in their motivation to interact with the different parts of the problem situation, we additionally coded the apes’ manipulation times with the available tools and the test apparatus and to perform some exploratory analyses. We coded the following durations per session: (i) manipulation of the apparatus (i.e., the horizontal tube) with hands, feet, mouth or with one of the tools, (ii) touching the distractor tools (stick, string), and (iii) touching the target tool (hose). Coding began when the chimpanzee entered the test room and ended upon success or after 300 seconds had elapsed. Behavioural categories were not mutually exclusive; for example, manipulation of the apparatus and touching a tool could overlap.

**Results.** In session 1, when the task was novel to both groups, there was no significant difference in the manipulation time of the apparatus (Welch-test on ranked data: *t* = 0.10, *df* = 12.97, *p* = 0.921). In the first session, there was a weak trend for the chimpanzees from the experience group to manipulate the distractor tools longer than chimpanzees from the control group did (Welch-test: *t* = -1.81, *df* = 13.81, *p* = 0.093). Finally, chimpanzees from the control group manipulated the hose significantly longer than the chimpanzees from the experience group in the first session (Welch-test: *t* = -2.42, *df* = 13.86, *p* = 0.030). All tests were performed with proportions. Absolute and relative means for each group are shown in Table S4.

**Table S4. Motivation to engage with the task per group in Study 1.** Absolute and relative manipulation times with the test apparatus, the distractor tools and the hose are listed per group and session (mean +/- SD).

|  |  | **Absolute [sec.]** | | | **Proportion [%]** | | |
| --- | --- | --- | --- | --- | --- | --- | --- |
| **Session** | **Group** | **Apparatus** | **Distractor tools** | **Hose** | **Apparatus** | **Distractor tools** | **Hose** |
| 1 | Exp | 59 (+/-58) | 61 (+/-62) | 16 (+/-32) | 41 (+/-27) | 46 (+/-32) | 10 (+/-11) |
| 1 | Ctrl | 29 (+/-32) | 21 (+/-30) | 20 (+/-22) | 45 (+/-20) | 26 (+/-28) | 29 (+/-22) |
| 2 | Exp | 20 (+/-22) | 14 (+/-23) | 14 (+/-27) | 28 (+/-28) | 13 (+/-28) | 28 (+/-32) |
| 2 | Ctrl | 16 (+/-16) | 10 (+/-13) | 14 (+/-15) | 30 (+/- 26) | 19 (+/-24) | 34 (+/-27) |

**Study 2**

**Methods. *Participants.*** Four chimpanzees had to be excluded from the study because they did not reliably drink from the drinking device during the prior experience phase (Table S5).

***Materials.*** A Plexiglas tube (H 25 cm, diameter: 6 cm) which was closed at the bottom and about 2 cm from the top served as drinking container. A hole (diameter: 2.5 cm) was either drilled into the top closure (experience group) or not (control group). The tubes were attached to Plexiglas plates (L 25 cm x W 14 cm x H 1 cm), which were attached to the mesh in the test room. From the outside, both tube versions looked the same. The straw tool consisted of a bent blue rigid hose (L 40 cm, outer diameter: 0.6 cm, inner diameter: 0.4 cm). The U-shaped Plexiglas tube (approximately L 14 cm x W 11 cm, tube diameter: 3 cm) was located between two horizontal Plexiglas plates (L 17 cm x W 12.5 cm x H 1 cm) which were connected to another vertical Plexiglas plate (L 37 cm x W 22.5 cm x H 1 cm) which was then attached to the mesh from outside the test room. The outer part of the tube was painted light blue to make the round shape visible. A fluted stick with red longitudinal lines (L 40 cm, diameter: 1 cm) and a yellow soft string (L 40 cm, diameter: 0.3 cm) served as distractor objects.

**Table S5.** Participants of Study 2.

| **Participant** | **Species** | **Sex** | **Age** | **Rearing** | **Role** |
| --- | --- | --- | --- | --- | --- |
| Bangolo | Chimp-A | Male | 8 | Mother | Participant |
| Kofi | Chimp-A | Male | 12 | Mother | Participant |
| Kisha^1^ | Chimp-A | Female | 13 | Mother | Participant |
| Lobo | Chimp-A | Male | 13 | Mother | Participant & Demonstrator |
| Tai | Chimp-A | Female | 15 | Mother | Participant |
| Lome | Chimp-A | Male | 16 | Mother | Participant & Demonstrator |
| Alex | Chimp-B | Male | 16 | Nursery | Participant |
| Bambari | Chimp-A | Female | 17 | Mother | Participant |
| Zira | Chimp-B | Female | 20 | Mother | Participant |
| Swela | Chimp-A | Female | 22 | Mother | Participant |
| Sandra | Chimp-A | Female | 24 | Mother | Participant |
| Frodo | Chimp-A | Male | 24 | Mother | Participant |
| Hope | Chimp-B | Female | 27 | Mother | Participant |
| Daza | Chimp-B | Female | 32 | Unknown | Participant |
| Dorien | Chimp-A | Female | 37 | Nursery | Demonstrator |
| Natascha^1^ | Chimp-A | Female | 37 | Nursery | Participant |
| Riet | Chimp-A | Female | 40 | Nursery | Participant |
| Fraukje^1^ | Chimp-A | Female | 41 | Nursery | Participant |
| Robert^1^ | Chimp-A | Male | 42 | Nursery | Participant |
| Frederike | Chimp-B | Female | 44 | Unknown | Demonstrator |

^1^ drop outs

***Procedure.*** The participant (test room 1) observed the demonstrator (test room 2) in the prior experience phase and later encountered the U-shaped tube in the test phase (test room 2). We provided the participant with a drinking device containing highly diluted grape juice to keep her in a place from which she could see the demonstrator. For the experience group, the drinking container was filled with pure grape juice; the straw was next to the container on the metal frame. As the participant drank in test room 1, the ape caregiver opened the door and the demonstrator entered test room 2. The experimenter called the demonstrator by name twice and knocked on the container, whereupon the caregiver left the test room area. The experimenter moved away from the chimpanzees to avoid distraction but remained in the test room area. When the demonstrator finished the juice, the experimenter returned and exchanged the straw for half a pellet. The caregiver was called in and lured the demonstrator to the corner of the test room with food. The experimenter refilled the tube and placed the straw on the metal frame. The caregiver released the demonstrator, and the procedure of the previous demonstration was repeated. For the control group, the drinking container was empty and blocked so that the straw could not be inserted. Instead of drinking the juice from the container, the demonstrator was free to handle the straw. The demonstrators manipulated the straw during some sessions, but not all. However, they all exchanged the straw with the experimenter after 30 seconds (i.e., they handled the tool). The first test session was conducted immediately after the last two demonstrations on the same day. The demonstrator left test room 2 and the experimenter set up the experimental setup (a dry drinking container and the U-shaped tube); then the participant entered test room 2. The second session was conducted on a different day. The same demonstrators were used for both groups. We cannot say for sure whether participants paid attention to the demonstrations, but at least their heads were turned toward the demonstrator.

***Coding and analyses.*** *Reliability.* A first coder (ASA) coded the videos and a second coder (SJE) recoded them; reliability between the two coders was good (Pearson’s correlation; time until finding the solution strategy: *r* = 1, *df* = 26, *p* < 0.001; time until tool extraction: *r* = 1, *df* = 24, *p* < 0.001; time until contact with tool: *r* = 1, *df* = 24, *p* < 0.001; Cohen’s Kappa; first tool use, *Κ* = 0.94, *N* = 28, *p* < 0.001 ; sucking attempt: *Κ* = 0.73, *N* = 28, *p* < 0.001). All analyses were performed in R-3.5.0^4^.
*Solution strategy.* We ran a GLMM with a binomial error structure and finding the solution strategy (yes/no) as the response (*N* = 28). The model included group and session as fixed effects and the random intercept of participant plus the random slope of session within participant as random effects. Session was standardized to its mean and served only as control variable. The model was treated in the same manner as the models in the previous study and showed the same instability, which was not surprising given the small sample size. Since session served only as a control variable and group was the only predictor tested, the comparison of the full and null model was equivalent to the test for the predictor group and is not reported.
*Time until solution strategy / tool extraction / contact with tool.* We ran Cox mixed models with the same fixed and random effects structure as the model with the solution strategy as the response (*N* = 28). The stability of these models looked reasonable.
*First tool use / Sucking attempts.* First, we ran GLMMs with a binomial error structure (*N* = 28). The models included the same fixed and random effects as the previous models but were highly unstable because most participants always scored the same. We therefore analysed the overall behaviour with a generalized linear model (GLM) that included group as a fixed effect (*N* = 14).

**Results. *Solution strategy.*** The results of the model are shown in Table S6.

**Table S6.** Results for the model with finding the solution strategy as the response.

| **Term** | **Estimate** | **SE** | ***χ^2^*** | ***df*** | ***p*** |
| --- | --- | --- | --- | --- | --- |
| Intercept | 0.961 | 0.655 | - | - | - |
| Group(Experience) | -1.926 | 1.009 | 5.23 | 1 | 0.022 |
| Session | -0.369 | 0.456 | ^1^ | ^1^ | ^1^ |

^1^ Session served as a control variable only.

***Time until solution strategy.*** The results of the model are shown in Table S7.

**Table S7.** Results for the model with time until finding the solution strategy as the response.

| **Term** | **Estimate** | **SE** | ***χ^2^*** | ***df*** | ***p*** |
| --- | --- | --- | --- | --- | --- |
| Group(Experience) | -1.220 | 0.682 | 3.37 | 1 | 0.066 |
| Session | -0.232 | 0.284 | ^1^ | ^1^ | ^1^ |

^1^ Session served as a control variable only.

**References S1 and S2**

1. Manrique, H. M. & Call, J. Spontaneous use of tools as straws in great apes. *Anim Cogn* **14**, 213–226 (2011).

2. Krupenye, C., Kano, F., Hirata, S., Call, J. & Tomasello, M. Great apes anticipate that other individuals will act according to false beliefs. *Science* **354**, 110–114 (2016).

3. Völter, C. J. & Call, J. Problem solving in great apes (*Pan paniscus, Pan troglodytes, Gorilla gorilla,* and *Pongo abelii*): the effect of visual feedback. *Anim Cogn* **15**, 923–936 (2012).

4. R Core Team. R: A language and environment for statistical computing. (2013).

5. Péter, A. Solomon Coder (version beta 12.09.04): A simple solution for behavior coding. (2011).

6. Bates, D., Maechler, M., Bolker, B., & Walker, S. lme4: Linear mixed-effects models using Eigen and S4. (2014).

7. Barr, D. J., Levy, R., Scheepers, C. & Tily, H. J. Random effects structure for confirmatory hypothesis testing: Keep it maximal. *Journal of Memory and Language* **68**, 255–278 (2013).

8. Schielzeth, H. & Forstmeier, W. Conclusions beyond support: overconfident estimates in mixed models. *Behavioral ecology* **20**, 416–420 (2009).

9. Therneau, T. M. coxme: Mixed Effects Cox Models. R package version 2.2-3. (2012).

10. Field, A. P. Is the meta-analysis of correlation coefficients accurate when population correlations vary? *Psychological methods* **10**, 444 (2005).

11. Fox, J., & Weisberg, S. *An {R} Companion to Applied Regression*. (Sage, 2011).

12. Mendes, N., Hanus, D. & Call, J. Raising the level: orangutans use water as a tool. *Biol. Lett.* **3**, 453–455 (2007).

13. Therneau, T. M. A package for survival analysis in S. R package version 2.38. (2015).

14. Therneau, T. M., & Grambsch, P. M. *Modeling survival data: extending the cox model*. (Springer, 2000).

15. Luchins, A. S., & Luchins, E. H. *Rigidity of behavior*. (University of Oregon Books, 1959).

16. Schultz, P. W. & Searleman, A. Rigidity of thought and behavior: 100 years of research. *Genetic, social, and general psychology monographs* **128**, 165 (2002).
