# Supplementary material for: Functional fixedness in chimpanzees
Source: Sci Rep. 2024 May 28;14:12155. doi: 10.1038/s41598-024-62685-w (PMC11130300; doi:10.1038/s41598-024-62685-w)
Supplement: Supplementary file 2 — Supplementary Information 2. [file 41598_2024_62685_MOESM2_ESM.docx]

**Functional fixedness in chimpanzees**

Ebel, Sonja J.; Völter, Christoph J.; Sánchez-Amaro, Alejandro; Helming, Katharina A.; Herrmann, Esther; Call, Josep

***Supplementary Material***

**Preliminary and follow-up studies**

**Preliminary studies**

**Prelim A: Brush study**

**Introduction.** The brush study was conducted before Study 1 and 2, but the results were not easy to interpret due to a floor effect. In this study, we examined functional fixedness in bonobos, chimpanzees and orang-utans. In the prior experience phase, the apes either dipped the brush end of a tool through a top opening into an apparatus filled with juice (experience group) or explored the tool without a task (control group). In the test, the dipping option was blocked and participants had to use the pointed end of the same tool to puncture a hole in the bottom of the apparatus to access the juice. Since the task and tool were the same in the prior experience phase and the test phase, this setup did not necessarily distinguish between a functional fixedness effect and an Einstellung effect (i.e., a fixation on the task or solution; also called ‘mental set’).

**Methods. *Participants.*** The final sample consisted of 23 great apes (5 bonobos, 12 chimpanzees, 6 orang-utans; Table S8). Three additional chimpanzees who did not dip on their own within the six shaping sessions in the prior experience phase were consequently excluded from the study. Additionally, one bonobo stopped dipping after 98 dipping events and was also excluded. Five chimpanzees from the final sample had prior experience with a brush-like tool: They had learned to prefer a wooden stick frayed at one end (i.e., brush-like) to an unmodified stick to obtain a food reward; some of these individuals had also manufactured such tools themselves by chewing on the stick (unpublished data). We distributed these individuals equally between the two groups to account for differences in prior experience. In addition, two chimpanzees had already handled a brush before, but they had not used it for dipping (see Prelim B).

***Materials*.** We used a Plexiglas tube filled with juice (H 17.5 cm; outer diameter: 9 cm), the upper opening of which was either left open (prior experience phase) or covered with a metal grid (test phase). A hole was drilled in the lower part of the tube (diameter: 0.9 cm) which was covered with a piece of adhesive tape from the inside of the tube. In the prior experience phase, the hole did not penetrate the second layer of Plexiglas, resulting in the release of juice only during the test phase (see Figure S1). The tube was filled with grape juice (chimpanzees, orang-utans: 120ml; bonobos: 100ml; we adjusted the amount of juice to match the additional food allowance set by the caregivers for each ape species). It was attached from the outside to the mesh of the apes’ sleeping rooms (hereafter: test rooms). For orang-utans, a thin tube (length: 9.5cm) was attached to the back of the apparatus during the test phase in case they would spit water into the tube^12^. However, they did not exhibit this behaviour. As a result of this adjustment, the grape juice amount was slightly reduced for orang-utans (100 ml). The wooden brush tool (about L 30 cm x W 2 cm x H 0.7 cm) comprised two functional ends: a brush end (about L 5 cm) and a pointed end (about L 2.5 cm).


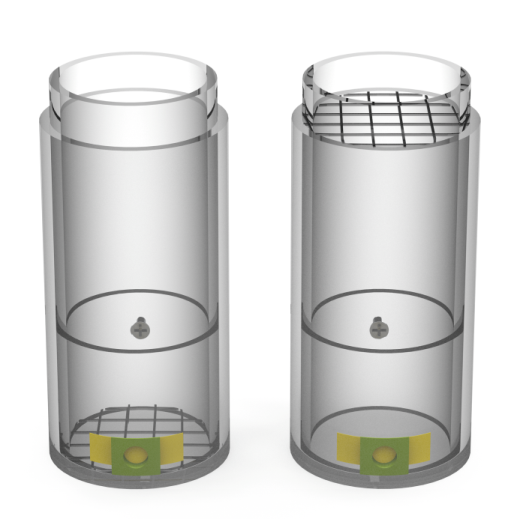


B

A

**Figure S1.** The apparatus in the brush study: In the prior experience phase (A), one group dipped juice with the brush end of a tool, while the other group did not. In the test phase (B), the dipping option was blocked so that the apes had to use the pointed end of the tool to puncture a hole in the lower part of the juice container.

**Table S8.** Participants of the brush study.

| **Participant** | **Species** | **Sex** | **Age** | **Rearing** |
| --- | --- | --- | --- | --- |
| Fimi | Bonobo | Female | 8 | Mother |
| Gemena | Bonobo | Female | 10 | Mother |
| Luiza | Bonobo | Female | 11 | Mother |
| Lexi | Bonobo | Female | 17 | Nursery |
| Yasa^1^ | Bonobo | Female | 19 | Mother |
| Kuno | Bonobo | Male | 19 | Nursery |
| Bangolo | Chimp | Male | 7 | Mother |
| Kofi | Chimp | Male | 11 | Mother |
| Kisha^1^ | Chimp | Female | 12 | Mother |
| Lobo | Chimp | Male | 12 | Mother |
| Tai | Chimp | Female | 14 | Mother |
| Lome | Chimp | Male | 15 | Mother |
| Swela^1^ | Chimp | Female | 20 | Mother |
| Frodo | Chimp | Male | 22 | Mother |
| Sandra | Chimp | Female | 23 | Mother |
| Dorien | Chimp | Female | 35 | Nursery |
| Natascha | Chimp | Female | 36 | Nursery |
| Riet | Chimp | Female | 38 | Nursery |
| Corrie^1^ | Chimp | Female | 39 | Nursery |
| Fraukje | Chimp | Female | 40 | Nursery |
| Robert | Chimp | Male | 40 | Nursery |
| Suaq | Orang | Male | 7 | Mother |
| Raja | Orang | Female | 13 | Mother |
| Padana | Orang | Female | 18 | Mother |
| Dokana | Orang | Female | 27 | Mother |
| Pini | Orang | Female | 28 | Mother |
| Bimbo | Orang | Male | 36 | Nursery |

^1^ drop outs

***Procedure.*** Both groups received a prior experience phase and a test phase. In the prior experience phase, the experience group (*N* = 11) learned to use the brush end of the tool to extract juice in an efficient manner. Sessions lasted 5 minutes or until the ape had finished the juice. The control group (*N* = 12) was given the same tool as the experience group, with no apparatus present (two sessions with 5 minutes each). These sessions served to control for the novelty of the tool. For the prior experience group, the position and orientation of the tool varied depending on the scaffolding procedure. Initially, participants found the tool with the brush end inserted into the drinking container. If participants dipped with the tool juice on their own, they continued to receive the baseline sessions until they reached the criterion for moving to the test phase. Baseline sessions consisted of the tool lying on a metal frame in the test room, with the direction of the brush end counterbalanced across sessions. If they did not dip juice with the tool, we tried three steps (each lasting two sessions) to facilitate the solution. First, participants received a second session with the brush tool already inserted into the tube. Second, the experimenter showed participants how to use the tool (i.e., she had them lick the juice-soaked brush and then reinserted it into the tube for participants to take it; this was sometimes done several times within a session). Third, participants were given a different brush in case their failure was due to the brush we used (this condition was only used in three chimpanzees who eventually dropped out of the study). The experience group moved to the test phase once they had completed 200 dipping events with the brush end of the tool in the baseline sessions. The number of dipping events varied between participants as they were allowed to finish the juice in a given session (mean: 7 sessions, range: 4-13; dipping with brush end: mean = 222 events, range = 200-261; dipping with pointed end: mean = 7 events, range = 0-29). We added a session with a bonobo (Luiza) because in her last session she dipped juice 17 times with the pointed end and we wanted to strengthen the function of the brush end of the tool. All sessions lasted five minutes, and one session was conducted per day. If the apes were still dipping with the tool after five minutes, they were allowed to finish the juice.
 In the test phase, the participants were presented with the same tube, but its upper opening was blocked by a grid. Now, participants had to puncture the tape covering the hole at the bottom front of the tube with the pointed end of the tool to access the juice. Participants were given two 5-minute sessions. If they only solved the task in the second session, they received a third one. One orang-utan (Padana) from the experience group succeeded in breaking the grid and dipping with the brush end of the tool after 211 seconds in the second session. We gave her two more sessions in which we replaced the grid with a plate of polyvinyl chloride (PVC) with holes inside. The orang-utan did not solve the task during the additional sessions.

***Coding and analyses.*** We coded whether participants solved the task and how long it took them. Our measure of survival time is composed of elapsed time and success. In addition, we measured the duration of manipulation by type (brush end, pointed end, hand or mouth touch) in three different areas of the tube (upper opening, lower opening, other areas). We calculated the relative manipulation time for each combination of manipulation type and tube area for all participants and sessions. A second coder (EB) coded 20 % of the videos to assess inter-observer reliability (Cohen’s Kappa: success: *Κ* = 1, *N* = 10, *p* = 0.002; Pearson’s correlation: survival time: *r* = 1, *df* = 8, *p* < 0.001; manipulations – all combinations of tube area and manipulation type combined: *r* = 0.99, *df* = 88, *p* < 0.001; dipping – brush end and pointed end combined: *r* = 1, *df* = 24, *p* < 0.001).
 All analyses were performed using R-3.0.2^4^. Success was analysed using a GLM with a binomial error structure (*N* = 23). The model included group and age as fixed effects. Age was first log-transformed and then z-transformed to obtain a mean of zero and a standard deviation of one. We derived VIFs that showed collinearity was not a problem. We performed a comparison of the full model with a reduced model that included only the intercept with a likelihood ratio test (LRT) to determine the overall effect of the predictors. In case of a significant difference between the two models, p-values for each predictor were determined with LRTs comparing the full model with the respective reduced model. Survival time was analysed with a survival model and included the same predictors as the model with success as the response (*N* = 23; R package “survival”)^13,14^. The treatment of the model was the same as in the previous one.
 Manipulation was analysed in a two-step approach because 51 % of a given combination of manipulation type and tube area was zero. First, we analysed the occurrence of manipulation as a binary variable (yes/no) for all combinations of manipulation type and area using a GLMM with a binomial error structure (*N* = 441). We then analysed the subset of data in which a manipulation occurred using a GLMM with a Gaussian error structure and the relative duration of the manipulation as the response (*N* = 214). Both models included the following fixed and random effects structure. We included the four-way interaction between group, manipulation type, tube area and age, but the model did not converge. Therefore, we included the three-way interaction between group, manipulation type and tube area, as well as age, species, sex, and session as fixed effects and the random slopes of area, manipulation type, and session within participant. The binomial model also included an offset term (log-transformed duration of session divided by 60) to account for varying session lengths. Age was log-transformed, and age and session were z-transformed to a mean of zero and a standard deviation of one. We evaluated VIFs for both models and found that there was no problem with collinearity. In addition, we assessed the normal distribution and homogeneity of the residuals for the Gaussian model by plotting the residuals, which looked acceptable. We also assessed model stability by examining the impact of excluding one level of random effect at a time for the estimates of the fixed effects. Model stability appeared to be reasonable. However, the Gaussian model was unstable when a particular chimpanzee (Lobo) was excluded because he was the only representative in one of the combinations of the three-way interaction (the Gaussian model was based on a subset of the data). We compared the full models with the corresponding reduced models that included only the random effect terms using LRTs. We then excluded non-significant interactions and determined p-values using LRTs (see model with success as response). To further investigate significant interactions, we releveled the respective factors (binomial model) or divided the data by one variable from the interaction, reran the model and evaluated the other variable(s) (Gaussian model).

**Results. *Success and survival time.*** The GLM with success as the dependent variable was not significant compared to the null model (LRT: *χ^2^* = 4.59*, df* = 2, *p* = 0.101). Fifty percent of the participants from the control group and 18% from the experience group solved the task (Figure S2A). More specifically, four participants from the control and one from the experience group solved the task in the first session and two from the control and one from the experience group solved it in the second session. The model with survival time as the dependent variable showed a trend compared to the null model (LRT: *χ^2^* = 5.91*, df* = 2, *p* = 0.052). Participants from the control group tended to solve the task faster than participants from the experience group (LRT: *χ^2^* = 3.06*, df* = 1, *p* = 0.080; Figure S2B). In addition, younger participants tended to solve the task faster than older participants (LRT: *χ^2^* = 3.14*, df* = 1, *p* = 0.076).

B

A

**Figure S2.** Success (A) and survival time (B) as a function of group in the brush study. The circles indicate individual performance, with larger circles indicating more individuals. A survival time of 600 seconds means that the task was not solved. ^P^ One participant (Padana) broke the apparatus before 600 seconds had elapsed and gained access to the juice without using the pointed end of the tool. She was given two more sessions with a more solid apparatus and then failed to solve the task.

Upper

Touch

Pointed end

Brush-end

Lower

Other

**Figure S3.** Relative manipulation time is shown as a function of group, tube area, and manipulation type in the brush study. The columns indicate the manipulation type (brush end, pointed end, touch) and the rows indicate the area of the tube (upper, lower, other).

***Manipulation: binomial model.*** First, we examined the occurrence of manipulation for all combinations of manipulation type (brush end, pointed end, hand or mouth touch) and the area of the tube (upper, lower, other). The full model fit the data significantly better than the null model (GLMM; LRT: *χ^2^* = 102.18*, df* = 22, *p* < 0.001). We first removed the non-significant three-way interaction between group, manipulation type, and tube area (LRT: *χ^2^* = 1.19*, df* = 4, *p* = 0.880) and the non-significant two-way interaction between group and manipulation type (LRT: *χ^2^* = 1.01*, df* = 2, *p* = 0.603) from the model. We obtained a significant interaction between group and tube area (LRT: *χ^2^* = 12.63*, df* = 2, *p* = 0.002). Further examination of the interaction revealed three main significant findings: First, participants from the experience group manipulated the upper area of the tube more frequently than participants from the control group, and more frequently than the other tube areas. Second, we found the opposite pattern for the lower area, i.e., participants from the control group manipulated this area more often than participants from the experience group. Third, participants from both groups manipulated the lower area less than the other two areas.
 The interaction between manipulation type and tube area was also significant (LRT: *χ^2^* = 40.66*, df* = 4, *p* < 0.001). Further examination of the interaction again revealed three main significant findings: First, for all three manipulation types, participants manipulated the upper area more frequently than the other two areas. Second, they manipulated the lower area most often with their hand or mouth, then, with the pointed end, and least often with the brush end of the tool. Third, they manipulated the lower area less frequently with the brush end of the tool compared to the other two areas. In addition, older participants manipulated the tube less than younger ones (LRT: *χ^2^* = 16.69*, df* = 1, *p* < 0.001), but manipulation time did not differ with respect to sex (LRT: *χ^2^* = 0.15*, df* = 1, *p* = 0.703). Overall, participants manipulated the tube less frequently over sessions (LRT: *χ^2^* = 6.80*, df* = 1, *p* = 0.009). We found a significant effect of species, with chimpanzees manipulating the tube more than bonobos and orang-utans (LRT: *χ^2^* = 7.08*, df* = 2, *p* = 0.029). However, the species factor was estimated with the age effect given because we did not include an interaction between species and age in the model and plotting the data did not reveal a clear effect. The age range of bonobos was smaller than that of chimpanzees and orangs-utans, and the duration of the session (included as an offset term) was shorter overall for chimpanzees than for the other two species. Thus, an occurrence (i.e., a “1”) had a higher weight in chimpanzees compared with bonobos and orang-utans, and a non-occurrence (i.e., a “0”) had a lower weight.

***Manipulation: Gaussian model.*** In a second step, we examined the duration of manipulation for all combinations of tube area and manipulation type. The comparison of the full with the null model was significant (GLMM; LRT: *χ^2^* = 100.31*, df* = 22, *p* < 0.001; Figure S3). We found a significant three-way interaction of group, tube area and manipulation type (LRT: *χ^2^* = 11.73*, df* = 4, *p* = 0.019). Participants from both groups manipulated the upper area longer than the other two areas and longer with both functional ends than with the hand or mouth. However, the prior experience group manipulated the upper area longer than the control group. The control group manipulated the lower area longer than the experience group. Regarding the types of manipulation, they manipulated the lower and the other area longer by hand or mouth than the experience group. None of the other predictors proved significant (age, species, session, sex).

**Discussion**. Apes who had used the brush end of a tool to extract juice from the top opening of a tube manipulated this area more frequently than naïve individuals after the task required them to use the pointed end of the tool to puncture a hole in the lower bottom of the tube. In contrast to the preference for the top opening shown by experienced participants, naïve participants explored the entire apparatus more fully, particularly with their hands and mouth. However, this difference in manipulation pattern between groups did not translate into differential success, perhaps due to an overall low success rate (35%), although there was a trend suggesting that naïve participants solved the task faster than experienced ones. In addition, younger participants tended to solve the task faster than older participants, and they manipulated the apparatus significantly more diversely (but not for a longer duration) than older participants.
 Although participants in the experience group manipulated the top opening of the tube more than the naïve participants, the former did not direct significantly more manipulations with the brush end of the tool to the (blocked) top opening, as would have been expected based on a functional fixedness effect. It is conceivable that participants in the experience group were fixated on the function of the tool but not specifically on the brush end of the tool: First, they manipulated the top opening significantly more often with the pointed end than participants in the control group. Second, 8/11 participants dipped the pointed end of the tool into the juice at least once during the prior experience phase, so that the tool as a whole may have acquired a dipping function. Moreover, it is possible that participants were fixated on the brush end of the tool, but that they were flexible enough to incorporate causal information into their judgements and understood that the brush end would not fit through the grid, while the pointed end would be useful in attempting to break the grid.
 Another possibility is that they persisted in their previous solution, commonly referred to as Einstellung effect or a mental set^15,16^. To separate the Einstellung effect from the functional fixedness effect, one would need to use two different tasks and/or allow participants to choose between at least two tools during the test. Thus, even if we had found a significant difference between groups, we would not have been able to separate the two fixation effects. In Study 1, we therefore used two different apparatus for the prior experience and the test phase. We also used a tool without differentiated functional parts (i.e., brush end vs. pointed end) to eliminate the possibility that participants perceived two different tools (corresponding to different functional parts). Our new tool consisted of a hose that could be used for drinking and for poking. These two functions were based on different physical properties of the tool (i.e., the hollowness and rigidity of the tool).

**Prelim B: L-shaped tool (pilot)**

**Introduction.** Originally, we designed a test with another tool with two functional ends which we tested before the brush study. The L-shaped tool could be used to rake in a container with a piece of food or to puncture a bag filled with juice. The pilot study is only briefly described here.

**Methods**

***(1) Bonobos & orang-utans, L-shape tool.****Participants.* 5 bonobos and 5 orang-utans participated in the study (Table S9).
*Materials.* We used an inclined metal plate, a magnetic container with a grape inside, an L-shaped piece of Plexiglas to which a bag of diluted grape juice was attached, and an L-shaped tool (L 35 cm, hook: 4 cm, diameter: 1 cm; Figure S4). For the new raking task (after overtraining, see below), we used a plate with an attached vertical rod that could be raked in on the metal plate guided by rails. Attached to the plate was a short tube containing a reward (banana pellet).
*Procedure.* The apes started with either the raking task (raking in a container with the L-end of the tool) or the puncturing task (puncturing a bag of juice with the pointed end of the tool). After that, they were given the other task (regardless of their performance). The apes were given three sessions to initially solve the task and then, had up to six sessions to complete 36 trials (with a maximum of 12 trials per session). The apes had a maximum of ten minutes to solve the task in each trial (otherwise the session was terminated), and the session had a maximum length of 30 minutes, i.e., no trial could be started after 30 minutes. If the apes bent or broke the tool or discarded it outside the test room, they were given the tool back or a new one. The trial began with the experimenter handing the tool to the ape in the correct orientation (i.e., with the pointed end in the rake task and the L-end in the puncture task). The tool was exchanged after the trial was completed. Since most apes could not solve the raking task, we formed pairs according to the number of successful trials in the puncturing task, divided the apes into two groups, and overtrained one group with the puncturing task (6 sessions with a maximum of 12 trials each). Both groups were given a new raking task (3 sessions with a maximum of 12 trials each). One orang-utan (Suaq) participated in the overtraining only but did not enter the test room for the new raking task afterwards.

***(2) Chimpanzees, L-shape tool, brush-tool.****Participants.* (a) Four chimpanzees (Fraukje, Kofi, Lome, Natascha) and (b) two chimpanzees (Lome, Fraukje) participated in the experiments respectively. *Materials.* (a) We adapted the materials from (1): The L-shaped tool was shortened in length (30cm) to make it easier to handle. The magnetic container was partially covered with a transparent piece of Plexiglas, ensuring that the apes were not distracted by attempting to directly extract the banana pellet with the tool (used here instead of a grape), and the magnetic force of the container was reduced; it was also placed closer to the mesh. (b) We used a dipping task instead of a raking task: The L-shaped tool had an L-shaped brush end and a pointed end. The brush end of the tool was sued to extract diluted grape juice from a horizontal tube located on the metal plate.
*Procedure.* One session of (a) the raking task and (b) the dipping task was performed, each lasting 10 minutes.


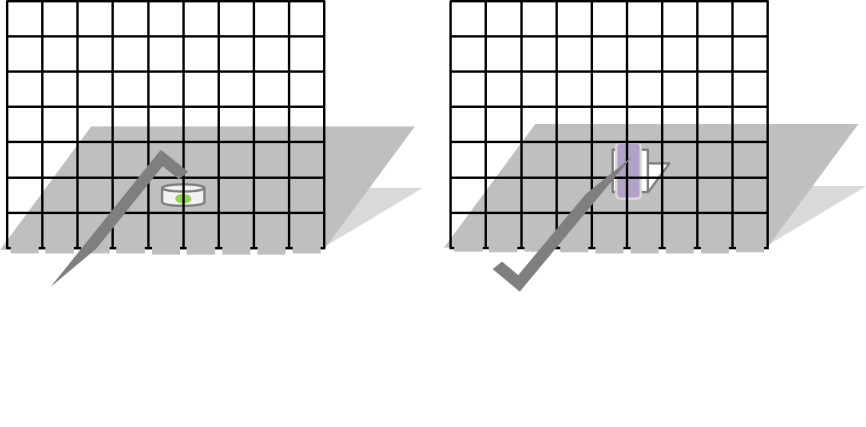
 **Figure S4.** The setup of the pilot study using an L-shaped tool with two functional ends. The L-shaped end could be used to rake in a magnetic container with a reward (A). The pointed end could be used to puncture a bag filled with juice so that the juice flowed out of the bag over the inclined metal board to the ape (B).

A

B

**Results.** Only 2/10 apes (Padana, Pini) raked in the container and another ape (Fimi) lifted the grape out of the container with the L-shaped end of the tool (Table S9). Because of this low success rate, we could not assess the influence of prior experience with the tool. Therefore, we divided the apes into two groups based on their performance in the puncturing task and overtrained one group with this task. However, the overtraining worked well only for two participants (Padana: 72/72 puncturing events, Luiza: 54/72), while the other apes lost motivation for the task (Gemena: 5/72; Kuno, Suaq: 2/72). Moreover, there was a floor effect in the new raking task, as only 3/9 solved it (Padana: 36/36 raking events; Pini: 24/36; Bimbo: 1/36). Two of these apes were from the control group and one from the overtrained group. None of the four additional chimpanzees tested with an adapted design of the raking task raked in the container. The two chimpanzees tested with an L-shaped brush tool and a dipping task showed no interest in the apparatus (time spent engaging with the apparatus – Fraukje: 0 seconds, Lome: 10 seconds).

**Table S9.** Results from the pilot study with an L-shaped tool.

| **Participant** | **Species** | **First task** | **Puncture – success** | **Rake – success** | **Puncture –  latency until first  success [sec.]** | **Rake –  latency until first  success [sec.]** |
| --- | --- | --- | --- | --- | --- | --- |
| Padana | Orang | Puncture | 36/36 | 36/36 | 50 | 290 |
| Gemena | Bonobo | Puncture | 25/36 | 0/36 | 636 | (1800)^3^ |
| Kuno | Bonobo | Puncture | 16/36 | 0/36 | 134.4 | (1800)^3^ |
| Suaq | Orang | Puncture | 5/36 | 0/36 | 1572 | (1800)^3^ |
| Bimbo | Orang | Puncture | 2/36 | 0/36 | 238 | (1800)^3^ |
| Pini | Orang | Rake | 36/36 | 36/36 | 49 | 397 |
| Fimi | Bonobo | Rake | 21/36 | 28/36^1^ | NA^2^ | 458 |
| Luiza | Bonobo | Rake | 36/36 | 0/36 | 30 | (1800)^3^ |
| Raja | Orang | Rake | 31/36 | 0/36 | 126 | (1800)^3^ |
| Jasongo | Bonobo | Rake | 10/36 | 0/36 | 162 | (1800)^3^ |

^1^ Fimi extracted the grape with the L-shaped end of the tool. ^2^ Due to technical problems we did not have a videotape of the first session. ^3^ No success within three sessions (each lasted 600 seconds).

**Discussion.** In this study, we were not able to assess the impact of prior tool experience because both tasks did not work well and especially the raking task was only solved by a few participants. The magnet of the raking container might have been too strong, so that the container might have been too difficult to move. Nevertheless, apes sometimes pushed it away, so at least they had the experience that it could be moved. Also, the apes were able to touch the grape with the tool, so they tried to extract it out of the container rather than trying to rake in the entire container. In addition, the puncturing task was difficult for some participants because the bag containing the juice was movable and therefore, difficult to puncture. In addition, it contained only a small amount of juice, which was diluted to meet the allowed food intake limits for the experiments, as we planned twelve trials per session. This caused some participants to lose motivation in this task. The apes also manipulated both apparatus with both tool ends and were sometimes successful with the wrong end (e.g., when the bag of juice was already punctured, they sometimes squeezed it with the L-shaped end of the tool). Since adjusting the raking task did not improve the apes’ performance and the setup with the L-shaped brush tool did not work, perhaps because the apparatus was too far away from the apes and the juice too diluted, we changed the setup completely (see brush study above).

**Follow-up studies**

**Follow-up A: The role of context in Study 1**

**Introduction.** In Follow-up A, we addressed two questions that could not be answered in Study 1. First, we investigated whether the presence of the drinking container during the test had an effect on functional fixedness. It is possible that the apes from the experience group in Study 1 paid more attention to the hose for juice consumption than the apes from the control group, and the drinking container promoted this bias. Therefore, they may have paid less attention to the new task, resulting in poorer performance in this group compared with the control group. Therefore, we removed the drinking container during the test in Follow-up A. Second, the apes in Study 1 may have been fixated on the reward (juice) rather than the function of the tool and therefore searching for the juice during the test phase, which may have contributed to their longer latencies in solving the task. We therefore used a small container (“kinderegg”) filled with juice as a reward in Follow-up A. In addition, we changed the appearance of all experimental materials in terms of shape, color and/or haptic compared to the materials used in Study 1. The procedure of Follow-up A was essentially the same as in Study 1. Since we did not have access to naïve chimpanzees, we tested the same chimpanzees as before and kept the distribution between the two groups from Study 1. We also made the same hypotheses as in Study 1: Participants from the experience group would perform worse in the new task than participants from the control group. This study had been pre-registered prior to data collection (osf.io/ktcp6).

**Methods. *Participants.*** The aim was to test the same participants as in Study 1 (subject to availability and willingness of the apes to participate). However, two chimpanzees (Kofi, Bangolo) had left the zoo and could not be tested at the new zoo at the time of data collection (*N_final_* = 14). Group assignment was maintained as in Study 1 because experience was critical in this study (and naïve individuals were not available; Table S10).

***Materials.*** The appearance of the hose, drinking container, horizontal tube and the distractor tools differed from the those used in Study 1 and Study 2 (Figure S5). In addition, we used a kinderegg (i.e., a small yellow container) filled with grape juice as a reward in the test.

***Procedure.*** The procedure was the same as in Study 1: During the prior experience phase, the experience group received five sessions of drinking juice with a hose (5 minutes or until the juice was empty). The control group received two sessions to explore the tool (5 minutes). In the test, participants from both groups watched how the experimenter filled a kinderegg with juice and placed it inside the horizontal tube. Then, they had 5 minutes to retrieve the reward from the tube. They were presented with the target tool (hose) and two distractor tools (stick, string). The drinking box was not present during the test. Two test sessions were conducted on different days. If the apes solved the task in the second session, they received a third one (*N* = 2; these two sessions were not included in the analyses as in Study 1).

***Coding & Analyses.*** The same behavioural measurements from the videos were coded and the same analyses were performed as in Study 1.

**Study 1**

**Follow-up A**


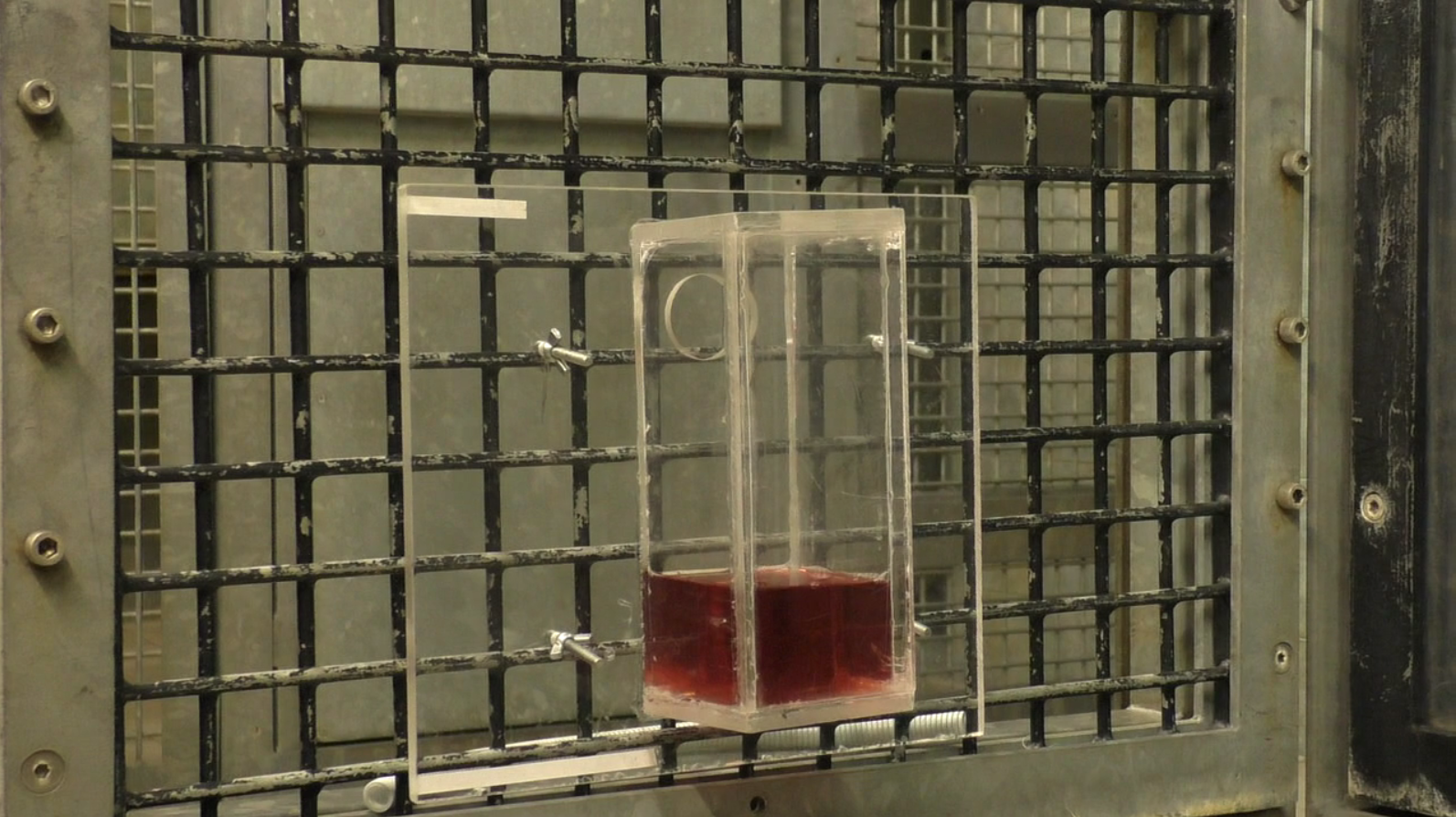

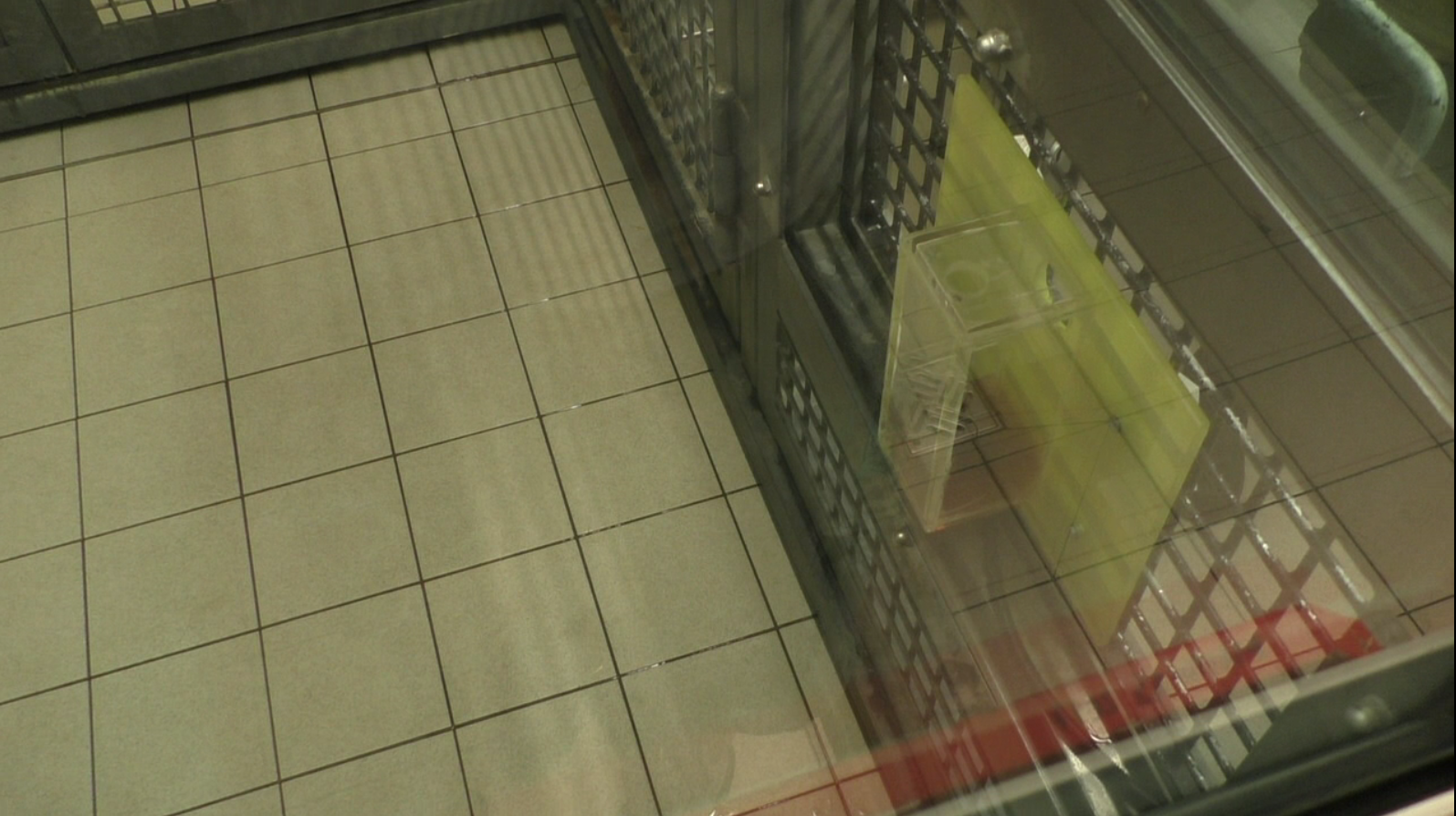


round tube, pellet^1^ as reward, lateral grey blockades

rectangular tube, juice inside a kinderegg, lateral blue blockades

B

A

inside of test room, hole at top, green back panel

outside of test room, hole at front, transparent


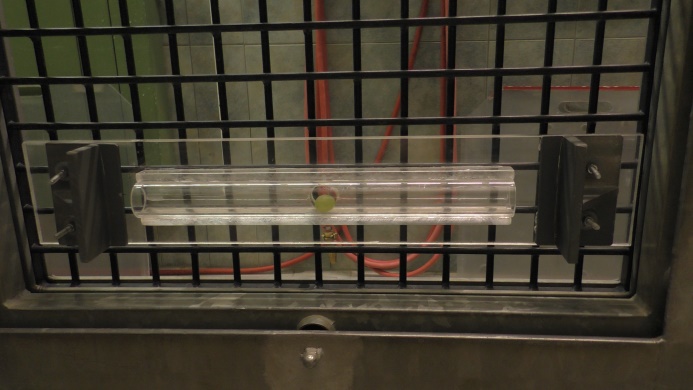

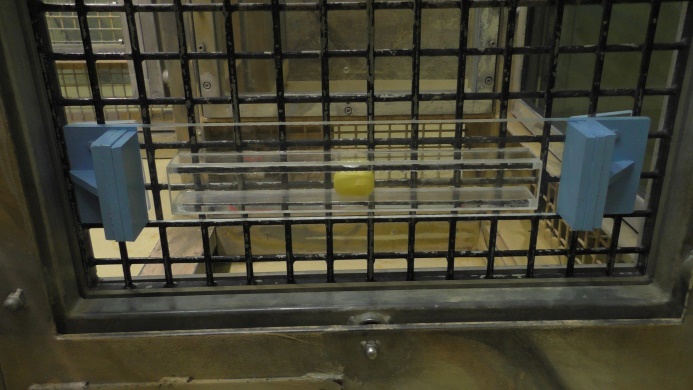


drinking container present, grey hose inserted into the container, wooden stick, white string

D

C


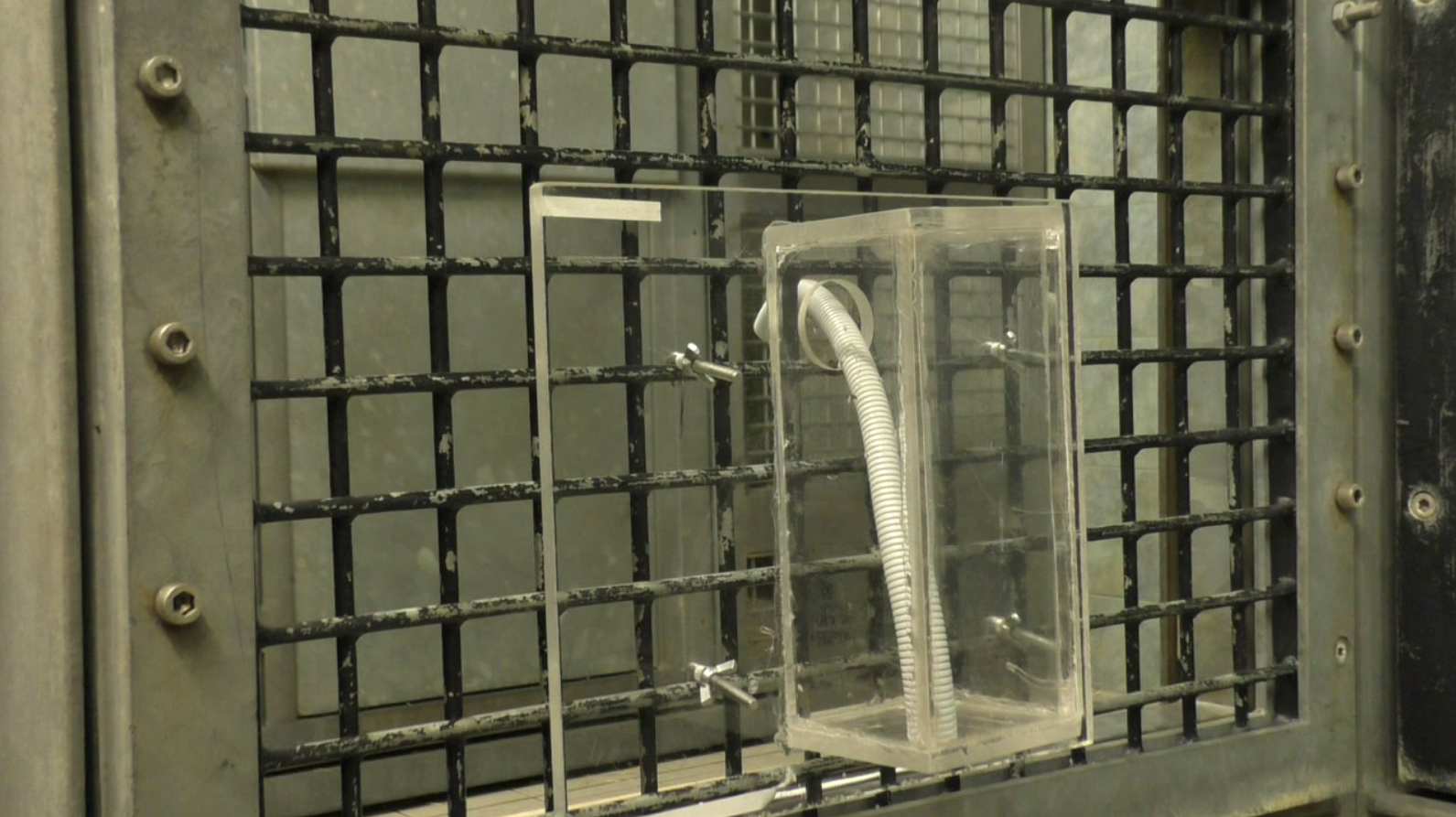

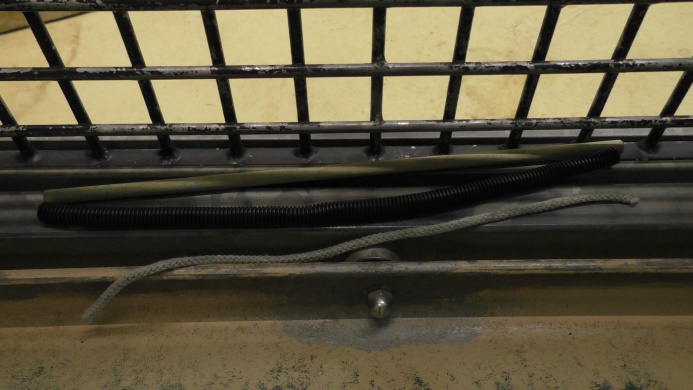


drinking container absent, black hose next to other tools, green stick, grey string

F

E

**Figure S5.** The setup of Follow-up A (B, D, F): The chimpanzees were presented with the same experimental setup as in Study 1 (A, C, E). However, first, the drinking container was removed during the test. Second, the food reward was the same (juice) in the prior experience phase and in the test. ^1^ Although a grape is shown in the photograph (which has never been used with the apes), a banana pellet was used in the original test.

**Results.** No difference was found in success rates between the two groups (Fisher’s exact test: *N* = 14, *p* = 1; Exp: 6/7, Ctrl: 6/7). Survival time until success showed only faster success across sessions, but no difference between groups (Cox Mixed Model; Likelihood ratio test [LRT] for full-null-model comparison: χ^2^ = 6.58, df = 2, *p* = 0.037; LRT for group: χ^2^ = 1.81, df = 1, *p* = 0.178; LRT for session: χ^2^ = 5.200, df = 1, *p* = 0.023; Figure S6A). The groups did not differ in how long it took them to touch the hose for the first time (Cox Mixed Model; LRT for full-null-model comparison: χ^2^ = 1.74, df = 2, *p* = 0.418; Figure S6C) or to remove it from the metal frame (Cox Mixed Model; LRT for full-null-model comparison: χ^2^ = 2.04, df = 2, *p* = 0.360; Figure S6B). There was also no difference between groups in the number of first attempts with the hose in session 1 (Fisher’s exact test: p = 0.559; Exp: 1/7, Ctrl: 3/7) or in the occurrence of sucking attempts between groups (Fisher’s exact test: p = 0.266; Exp: 4/7, Ctrl: 1/7). Table S9 provides an overview of participants’ performance in terms of their drinking and poking experience with hoses in Study 1, Study 2 and Follow-up A.

**Table S10.** Participants’ experience of successful drinking and poking with the tool (hose) in Study 1, Study 2 and Follow-up A.

| **Participant** | **Population** | **Study 1** | | **Study 2** | | **Follow-up A** | | **Notes** |
| --- | --- | --- | --- | --- | --- | --- | --- | --- |
|  |  | **Drinking** | **Poking** | **Drinking** | **Poking** | **Drinking** | **Poking** |  |
| Bangolo | A-chimps | Yes | Yes | Observe | No | - ° | - ° | ° had left the zoo |
| Dorien | A-chimps | Some drinking (dropout) | - | Yes ^+^ | No | - | - | ^+^ Demonstrator in Study 2 |
| Fraukje | A-chimps | Yes | No | Some observing (dropout) | - | Yes | No |  |
| Frodo | A-chimps | No | Yes ^ | Observe | No | No | No | ^ solved only in S1 & then lost interest |
| Kisha | A-chimps | - | - | No (dropout) | - | - | - |  |
| Kofi | A-chimps | No | Yes | No | No | - ° | - ° | ° had left the zoo |
| Lobo | A-chimps | Yes | Yes | No + Yes ^+^ | No | Yes | Yes | ^+^ First participant, then demonstrator in Study 2 |
| Lome | A-chimps | No | Yes | Observe + Yes ^+^ | No | No | Yes | ^+^ First participant, then demonstrator in Study 2 |
| Natascha | A-chimps | - | - | Some observing (dropout) | - | - | - |  |
| Riet | A-chimps | No | Yes | No | No | No | Yes |  |
| Robert | A-chimps | Yes | No | No (dropout) | - | Yes | Yes |  |
| Sandra | A-chimps | No | Yes | Observe | No | No | Yes |  |
| Swela | A-chimps | No | Yes | No | No | No | Yes |  |
| Tai | A-chimps | Yes | Yes | No | Yes | Yes | Yes |  |
| Alex | B-chimps | Yes | No | Observe | No | Yes | Yes |  |
| Bambari | B-chimps | No | Yes | Observe * | No * | No * | Yes * | * lived in A-chimp group at the time of testing |
| Daza | B-chimps | Yes | Yes | Observe | No | Yes | Yes |  |
| Frederike | B-chimps | No | Yes | Yes ^+^ | No | No | Yes | ^+^ Demonstrator in Study 2 |
| Hope | B-chimps | Yes | Yes | No | No | Yes | Yes |  |
| Zira | B-chimps | - * | - * | No | No | - | - | * lived in A-chimp group at the time of testing |

**
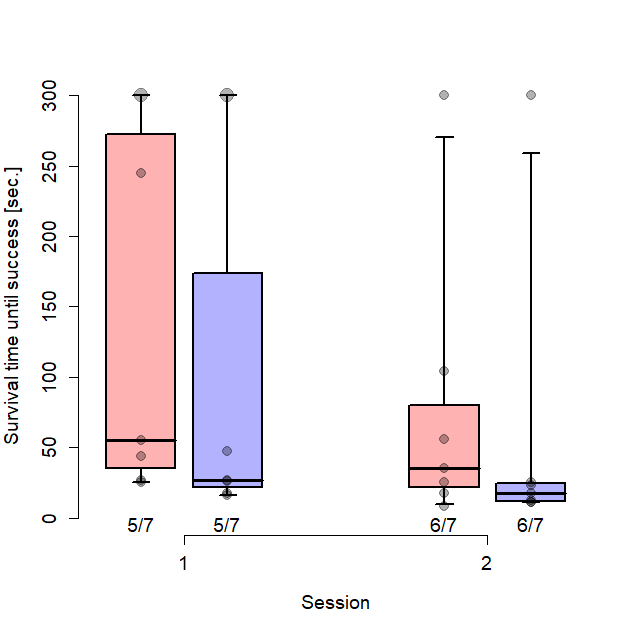

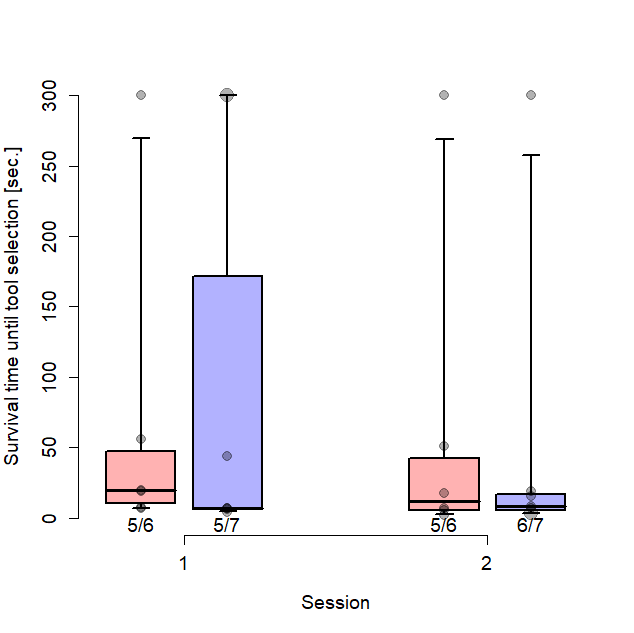
** **
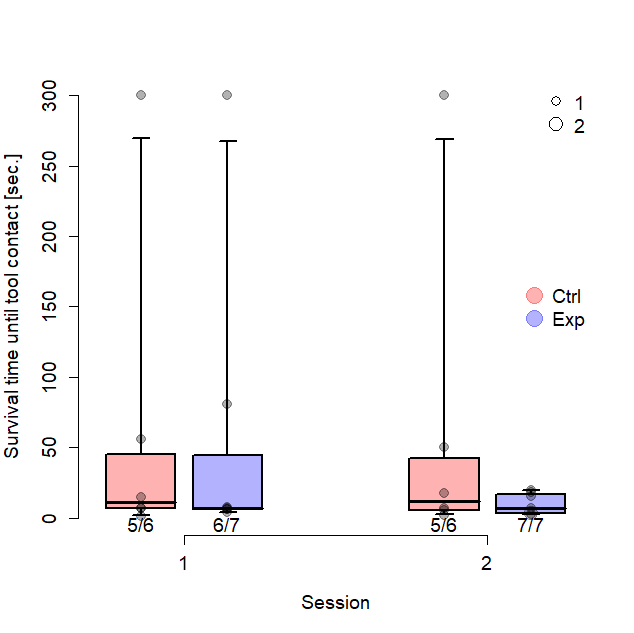
**

C

B

A

**Figure S6.** Results of Follow-up A: (A) survival time until success (or until the end of the session at 300 sec; survival time is a compound of success and time), (B) survival time until target tool extraction and (C) latency until target tool contact across groups and sessions (median; boxes: 0.25, 0.75; whiskers: 0.025, 0.975). Circles designate individual performance; the circle size is proportional to number of individuals represented.

**Discussion.** Repeating the same paradigm from Study 1, except that the drinking container was removed and the reward pellet was replaced with a small container filled with juice, revealed no significant difference between the two groups. That is, the chimpanzees who had been drinking juice with a hose before the test were not slower at solving the new task, which required them to use the hose to poke. However, the results are confounded in two ways. First, participants had solved a comparable poking task in Study 1 and therefore, were not naïve in the test. Second, assuming that the change in experimental design reduced the fixation effect (and not the experience of the control participants in Study 1), it remains unclear whether removing the drinking container or replacing the reward in the test phase (juice) caused the effect to disappear.

**Follow-up B: The pre-utilization condition**

**Introduction.** In Follow-up B, we examined the role of context on the functional fixedness effect with a different experimental design. The experimental design was more in line with the designs used in developmental research, where the tool is presented in its functional context, referred to as “pre-utilization condition” (e.g., a spoon in a pot of rice vs. the spoon next to the pot, or a box filled with objects vs. a box and objects lying next to each other). We conducted two tests with chimpanzees: (a) They were either presented with raisins in a bucket that was closed with a lid, or the raisins were presented next to the bucket and the lid. Then, the chimpanzees had to use the lid to solve a new task. (b) The chimpanzees were either presented with yoghurt in a yoghurt pot or the yoghurt was placed next to the pot. Then, the chimpanzees had to use the yoghurt pot to solve a new task. Follow-up B was pre-registered before data collection (osf.io/6fth5).

**Methods.** ***Participants.*** We tested all chimpanzees available at WKPRC (*N* = 23; 16 females; mean age = 28 years, SD = 14, range = 3-53). One chimpanzee infant did not participate in the lid task because his mother managed to enter the test room through the partially open hydraulic door. The door was open so that the infant could reach for his mother at any time. Two other participants dropped out because they did not open the raisin bucket and/or did not eat the raisins. Finally, two participants could not be tested due to testing time constraints. Four of these participants were in the pre-utilization group and one in the control group (cup task: *N* = 23; lid task: *N_final_* = 18).

***Materials*.** *Pot task.* A yoghurt pot was filled with a spoonful of yoghurt (pre-utilization group; Figure S7B) or the yoghurt was placed next to the pot (control group; Figure S7A). The task consisted of two horizontal plates with a gap between them. Using a piece of plastic from the yoghurt pot, the apes could retrieve two pellet halves. The yoghurt pot was cut in two places to facilitate its usage.
*Lid task.* In this zoo, raisins were stored in large yoghurt buckets as a reward for the apes. For the test, an empty yoghurt bucket was filled with 30 raisins (pre-utilization group; Figure S7D) or the raisins were placed next to the bucket (control group; Figure S7C). For the pre-utilization group, the lid was placed on the bucket after participants observed the experimenter fill it with raisins. For the control group, the lid was placed under the bucket. The task consisted of two vertical plates with a gap between them. Participants could use the lid to poke out the two pellet halves. In addition, the handle of the bucket could also serve as a tool.

***Procedure.*** Participants were tested with both tasks on different days. The pot task was always presented first and the lid task second. For one task, participants were in the pre-utilization group, and for the other, they were in the control group. When they entered the test room, they had 5 minutes to complete the task. After that, they were released back into their social group.

***Coding & Analyses.*** We intended to analyse the data with GLMMs and Cox Mixed models (dependent on the dependent measures) but were unable to do so due to a floor effect. Therefore, we provide a descriptive account of the results. Additionally, due to the floor effect, we only report success rates and not survival times.


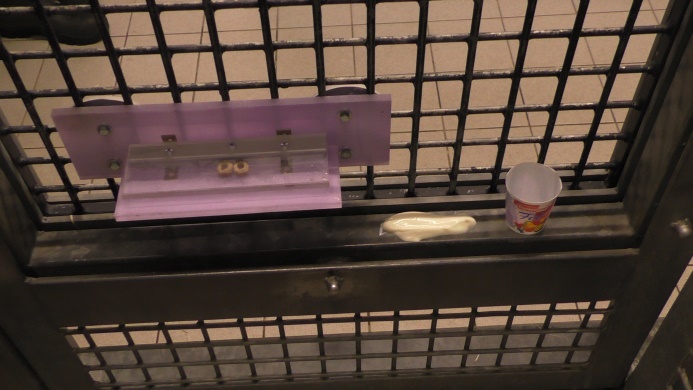

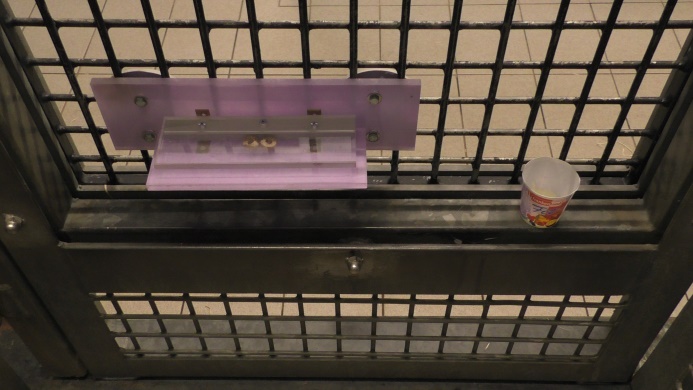


B

A


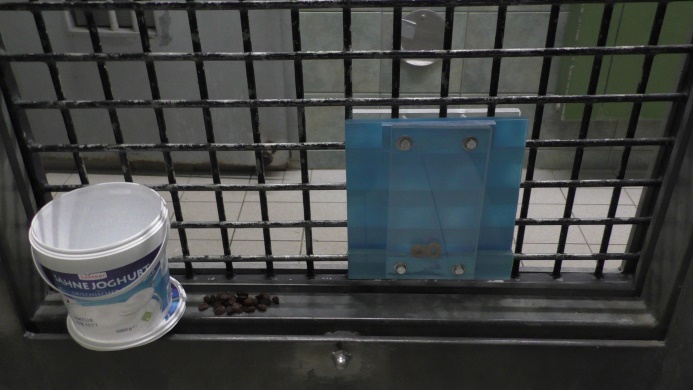

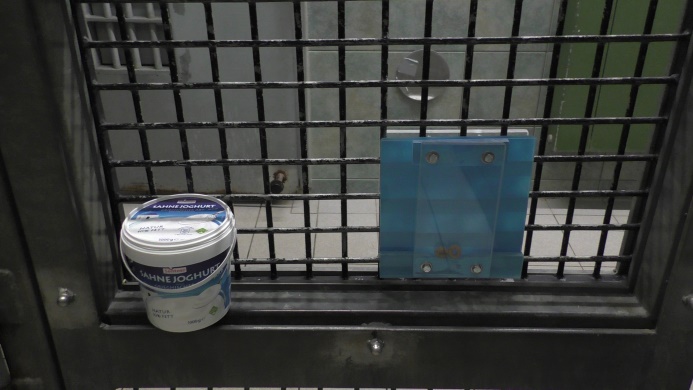


D

C

**Figure S7.** The setup of Follow-up B: In the pot task (A, B), the apes could retrieve pellets from the apparatus with a piece of plastic from the yoghurt pot. In the lid task (C, D), they could retrieve pellets from the apparatus with the lid of a raisin bucket (or its handle). The control group was presented with the tool next to the food reward (A, C), while the pre-utilization group was presented with the tools in their functional context, i.e., yoghurt inside the yoghurt pot (B) and raisins inside the raisin bucket which was closed with a lid (D). Note that the yoghurt pot was cut in two places to facilitate its use as a tool (A, B) and that the lid of the raisin bucket was placed under the bucket for the control group (C).

**Results*. Pot task.*** Overall, 25% (3/12) of the chimpanzees from the control group solved the task with a piece of plastic from the yoghurt pot compared to 0% (0/11) of the pre-utilization group (Table S11). However, some participants found an alternative solution: They extracted the pellet halves with their tongues. This was the case in 25% (3/12) of the control group and in 45% (5/11) of the pre-utilization group. Moreover, some chimpanzees found the correct solution strategy, i.e., they referred the yoghurt pot or a piece of it to the apparatus at least once without touching the pellets. This was not the case for any of the chimpanzees from the control group, but for 36% (4/11) of the chimpanzees from the pre-utilization group (of which 2/4 solved the task with their tongues afterwards).

***Lid task.*** None of the chimpanzees solved the task with the lid of the bucket (Table S11). Overall, 10% (1/10) of the chimpanzees from the control group solved the task with the handle of the bucket, whereas 50% (4/8) from the pre-utilization group did so. Moreover, some chimpanzees found the correct solution strategy with the lid, i.e., they referred the lid to the apparatus without touching the food reward. This was the case for 10% (1/11) of the control group and 25% (2/8) of the pre-utilization group (of which one participant from the pre-utilization group subsequently solved the task with the handle). The one participant from the control group who attempted to use the lid solved the task after 5 minutes with a single thread of wood wool.

**Table S11.** Results of Follow-up B. ^a^ Two of these apes were afterwards successful with their tongues. ^b^ The ape was afterwards successful with a single thread of wood wool. ^c^ The ape was afterwards successful with the handle.

| **Task** | **Group** | **Success with**  **pot** | **Success with**  **tongue** |  | **Referred pot to apparatus  (without success)** |
| --- | --- | --- | --- | --- | --- |
| Pot task | Control | 25% (3/12) | 25% (3/12) |  | 0% (0/12) |
|  | Experimental | 0% (0/11) | 45% (5/11) |  | 36% (4/11)^a^ |
|  |  |  |  |  |  |
|  |  | **Success with lid** | **Success with handle** | **Success with  wood wool** | **Referred lid to apparatus  (without success)** |
| Lid task | Control | 0% (0/10) | 10% (1/10) | 10% (1/10) | 10% (1/10)^b^ |
|  | Experimental | 0% (0/8) | 50% (4/8) | 0% (0/8) | 25% (2/8)^c^ |

**Discussion.** With the experimental design used, no difference was found between the pre-utilization and the control group. However, we consider this approach fruitful and future studies could use paradigms that induce function by presentation. At this point, we will focus on only one aspect in the discussion. Although the objects were somewhat familiar to the apes, they were usually controlled by humans in apes’ everyday life, and their own experience or familiarity might play a key role in the functional fixedness effect. Therefore, it would be more comparable to human studies to use a tool that the apes use in their everyday life in a specific context. Additionally, one could (if possible) use a problem that has been shown to be easily solved by a majority of the chimpanzees to avoid a floor effect. Finally, it might be useful to introduce distractor objects to reinforce the importance of tool selection for success in the task.

**References S1 and S2**

1. Manrique, H. M. & Call, J. Spontaneous use of tools as straws in great apes. *Anim Cogn* **14**, 213–226 (2011).

2. Krupenye, C., Kano, F., Hirata, S., Call, J. & Tomasello, M. Great apes anticipate that other individuals will act according to false beliefs. *Science* **354**, 110–114 (2016).

3. Völter, C. J. & Call, J. Problem solving in great apes (*Pan paniscus, Pan troglodytes, Gorilla gorilla,* and *Pongo abelii*): the effect of visual feedback. *Anim Cogn* **15**, 923–936 (2012).

4. R Core Team. R: A language and environment for statistical computing. (2013).

5. Péter, A. Solomon Coder (version beta 12.09.04): A simple solution for behavior coding. (2011).

6. Bates, D., Maechler, M., Bolker, B., & Walker, S. lme4: Linear mixed-effects models using Eigen and S4. (2014).

7. Barr, D. J., Levy, R., Scheepers, C. & Tily, H. J. Random effects structure for confirmatory hypothesis testing: Keep it maximal. *Journal of Memory and Language* **68**, 255–278 (2013).

8. Schielzeth, H. & Forstmeier, W. Conclusions beyond support: overconfident estimates in mixed models. *Behavioral ecology* **20**, 416–420 (2009).

9. Therneau, T. M. coxme: Mixed Effects Cox Models. R package version 2.2-3. (2012).

10. Field, A. P. Is the meta-analysis of correlation coefficients accurate when population correlations vary? *Psychological methods* **10**, 444 (2005).

11. Fox, J., & Weisberg, S. *An {R} Companion to Applied Regression*. (Sage, 2011).

12. Mendes, N., Hanus, D. & Call, J. Raising the level: orangutans use water as a tool. *Biol. Lett.* **3**, 453–455 (2007).

13. Therneau, T. M. A package for survival analysis in S. R package version 2.38. (2015).

14. Therneau, T. M., & Grambsch, P. M. *Modeling survival data: extending the cox model*. (Springer, 2000).

15. Luchins, A. S., & Luchins, E. H. *Rigidity of behavior*. (University of Oregon Books, 1959).

16. Schultz, P. W. & Searleman, A. Rigidity of thought and behavior: 100 years of research. *Genetic, social, and general psychology monographs* **128**, 165 (2002).
