# Supplementary material for: Functional fixedness in chimpanzees
Source: Sci Rep. 2024 May 28;14:12155. doi: 10.1038/s41598-024-62685-w (PMC11130300; doi:10.1038/s41598-024-62685-w)
Supplement: Supplementary file 6 — Supplementary Legends. [file 41598_2024_62685_MOESM6_ESM.docx]

41598_2024_62685_MOESM4.mp4

The video shows a participant from the control group during the test in Study 1.

41598_2024_62685_MOESM5.mp4

The video shows a participant from the experimental group during the test in Study 1.
